# Notch Stimulates Both Self-Renewal and Lineage Plasticity in a Subset of Murine CD9^High^ Committed Megakaryocytic Progenitors

**DOI:** 10.1371/journal.pone.0153860

**Published:** 2016-04-18

**Authors:** Michèle Weiss-Gayet, Joëlle Starck, Azza Chaabouni, Bénédicte Chazaud, François Morlé

**Affiliations:** 1 Institut NeuroMyoGène (INMG), Université Claude Bernard Lyon1, Villeurbanne, France; 2 INSERM U1217, Villeurbanne, France; 3 CNRS UMR 5310, Villeurbanne, France; Emory University, UNITED STATES

## Abstract

This study aimed at reinvestigating the controversial contribution of Notch signaling to megakaryocytic lineage development. For that purpose, we combined colony assays and single cells progeny analyses of purified megakaryocyte-erythroid progenitors (MEP) after short-term cultures on recombinant Notch ligand rDLL1. We showed that Notch activation stimulated the SCF-dependent and preferential amplification of Kit^+^ erythroid and bipotent progenitors while favoring commitment towards the erythroid at the expense of megakaryocytic lineage. Interestingly, we also identified a CD9^High^ MEP subset that spontaneously generated almost exclusively megakaryocytic progeny mainly composed of single megakaryocytes. We showed that Notch activation decreased the extent of polyploidization and maturation of megakaryocytes, increased the size of megakaryocytic colonies and surprisingly restored the generation of erythroid and mixed colonies by this CD9^High^ MEP subset. Importantly, the size increase of megakaryocytic colonies occurred at the expense of the production of single megakaryocytes and the restoration of colonies of alternative lineages occurred at the expense of the whole megakaryocytic progeny. Altogether, these results indicate that Notch activation is able to extend the number of divisions of MK-committed CD9^High^ MEPs before terminal maturation while allowing a fraction of them to generate alternative lineages. This unexpected plasticity of MK-committed progenitors revealed upon Notch activation helps to better understand the functional promiscuity between megakaryocytic lineage and hematopoietic stem cells.

## Introduction

Notch signaling is involved in many proliferation/differentiation and/or lineage commitment decisions during development, including hematopoiesis [1–3]. Notably, Notch1 is required for the generation of the first definitive hematopoietic stem cells (HSC). Notch1 is also required for T-cell lineage development occurring at the expense of B-cell lineage [4]. Moreover, deregulated Notch signaling induces T-cell leukemia in mouse and human [5].

Concordant *in vitro* results have shown that stimulation by Notch ligands (JAG1, JAG2, DLL1 or DLL4 [6–12] as well as enforced expression of Notch intracellular domain (NICD) [7] or that of its target HES1 [13] stimulate HSC self-renewal at the expense of their differentiation [14]. In apparent contradiction, most *in vivo* studies have shown that the steady state number of HSCs is not affected by the suppression of Notch signaling by either conditional deletion of Notch1[15], Notch2 [16], Notch1 and Notch2 [17], RBP-Jk [18], Jag1 [19] or Hes1 nor by enforced expression of the pan-Notch inhibitor dnMAML [20]. However, deletion of Notch2 (but not Notch1) reduces the rate of bone marrow reconstitution including repopulation of HSCs after injury thus suggesting a specific role for Notch2 during stress hematopoiesis [16].

Whether Notch also controls lineage commitment and/or self-renewal divisions of multipotent and/or committed monopotent progenitors still remains more controversial. Recent studies showed that Notch activation induces selective apoptosis of granulo-monocytic progenitors (GMPs) [21] while loss of Notch signaling induces myelo-monocytic leukemia in mouse and chronic myelo-monocytic leukemia (CMML) in human [21–25]. On the opposite, other studies have shown that Notch activation increases the number of CD41^+^ megakaryocytic cells generated by murine Lin^-^/Sca-1^+^/c-Kit^+^ (LSK), common myeloid progenitors (CMPs) or MEPs indicating the positive contribution of Notch to the megakaryocytic specification [26]. Further studies have shown that Notch pathway activates AKT that in turn suppresses the inhibitory action of FOXO factors on Notch targets during megakaryocytic development particularly in CMPs [27]. In both of these studies [26, 27], the positive effect of Notch on megakaryocytic development was systematically associated with increased numbers of MEPs and decreased numbers of GMPs that were interpreted as the successive contributions of Notch to the megakaryocytic commitment of CMPs and MEPs. However, intriguingly, Notch does not promote megakaryocytic commitment of human CD34^+^ pluripotent cells but inhibits terminal megakaryocyte maturation in contrast to what is observed in mouse [28]. These discrepancies were tentatively attributed to differences in the contribution of Notch to the control of megakaryocytic lineage between mouse and human [29]. Similarly, contradictory results have also been reported regarding the role of Notch during erythropoiesis with some studies indicating increased apoptosis [30, 31] and many others indicating either inhibition of erythroid differentiation [32–35] and/or increased self-renewal of committed erythroid progenitors [35, 36].

The aim of this study was to reinvestigate the real impact of Notch signaling in megakaryocytic lineage development. For this purpose, we took advantage of purified bipotent MEPs, which offer the precise deciphering of megakaryocytic commitment, expansion and differentiation through progeny analysis. Moreover, to avoid the side effects associated with the use of DLL1-expressing cells, MEPs were shortly activated *in vitro* with recombinant Notch ligand rDLL1.

## Material and Methods

### Mice

Mice (genetic background C57BL/6J-129) were bred and maintained under specific-pathogen-free conditions at the ALECS-SFP animal facility of the Faculté de Médecine Lyon-Est (Université Claude Bernard, Lyon1, France) and experimentations were performed according to procedures approved by the local animal care and experimentation authorities (Ministère Délégué de la Recherche et des Nouvelles Technologies, agreement no. 4936; Direction des Services Vétérinaires, agreement n°69266317 and 7462).

### Cell sorting and phenotypic characterization by flow cytometry

Bone marrow cells (BMC) were flushed from femurs and tibiae in Iscove's Modified Dulbecco's Medium (IMDM) containing 2% fetal calf serum (FCS), treated with red blood cells ACK lysing buffer (Lonza) and filtered through a 40 μm cell strainer (BD Biosciences) in order to obtain single-cell suspensions. BMC suspensions were labeled with a cocktail of biotinylated lineage antibodies (Lineage cell depletion Kit, Miltenyi Biotec) supplemented with biotinylated anti-Sca-1 (BD Pharmingen), anti-CD3 (BD Pharmingen), anti-IL7R (eBiosciences), anti-Ter119 (eBiosciences) and anti-CD19 (Serotec). Lin^-^/Sca1^-^ cell suspensions were isolated by magnetic depletion of lineage and Sca-1 positive cells using LS columns (Miltenyi Biotec). For MEP preparation, Lin^-^/Sca1^-^ cells were further labeled with streptavidin-PE-Cy7 (BD Pharmingen) for the elimination of residual biotinylated stained cells and with anti-c-Kit-APC (BD Pharmingen), anti-CD34-FITC (eBiosciences) and anti-Fcγ-RII/III-PE (BD Pharmingen) antibodies and sorted with gating window Lin^-^/Sca1^-^/Kit^+^ Fcγ-RII/III^low^/CD34^low^ (S1 Fig) as previously described [37] using FACSAria cell sorter and DIVA software (BD Biosciences). MEP subsets expressing different levels of CD9 were sorted using anti-cKit-efluor 450 and anti-CD9-APC antibodies. Other antibodies used to characterize MEPs progeny were the following: anti-CD41-FITC (eBiosciences), anti-CD42b-PE and anti-CD11b-APC (eBiosciences). For CD9^High^ LSK (Lin^-^/Sca1^+^/Kit^+^) cells preparation, Lin^-^ cells were first isolated by magnetic depletion of lineage positive cells using LS column (Miltenyi Biotec) and the same depletion cocktail but without added biotinylated Sca-1 antibody. Lin^-^ cells were then further labeled with anti-c-Kit-efluor 450 (eBiosciences), anti Sca-1-PE (eBiosciences) and anti-CD9-APC (eBiosciences) antibodies followed by the sorting of CD9^High^ LSK cells using gating windows shown in S1 Fig.

### Batch cultures of MEPs on recombinant rDLL1 and control IgG1

Cultures were performed as previously described with minor modifications [10]. Briefly, wells of untreated culture plates were pre-coated for 1 h at 37°C with 10 μg/mL of goat F(ab’)2 anti-human IgG1 Fc specific (Rockland) and of 25 μg/mL Retronectin (Takara) in phosphate buffered saline (PBS). Wells were washed twice with PBS, blocked with 1% bovine serum albumin (BSA) in PBS, incubated with 10 μg/mL of either IgG1 or rDLL1 (DLL1(mouse)-Fc(Human) chimeric recombinant protein, AG-40A-0148-C050, Adipogen) in PBS for 2 h at 37°C and washed extensively with PBS. Coated wells were seeded at day 0 with 2000 or 1000 progenitor cells in 1 mL of IMDM medium supplemented with 10% FCS, mIL3 (10 ng/mL), mSCF (30 ng/mL), mFlt3l (25 ng/mL), mGM-CSF (10 ng/mL), mIL11 (25 ng/mL), huEPO (4 U/mL) and mTPO (25 ng/mL) with or without 10 μM DAPT (γ-secretase inhibitor, Sigma) or 5 μg/mL of Notch2 neutralizing antibody (Clone B6, AB00176-1.1, Interchim) [38, 39]. At day 2, the totality of the cells from each well, representing the total progeny generated by the initial 1000 or 2000 cells seeded at day 0, were collected and analyzed by colony assay. Alternatively, cells were numbered and analyzed by flow cytometry after labeling with appropriate antibodies at day 5 or 6.

### Batch cultures of MEPs on OP9-DLL1 and control OP9 stromal cells

Control OP9 (OP9-GFP) and OP9-DLL1 stromal cells (kindly provided by Dr Thomas Mercher) were cultured in OP9 medium: α-MEM (StemCell technologies) containing 20% FCS, 50 μM 2-mercapto-ethanol, 2 mM glutamine (GIBCO), 0.2% sodium bicarbonate (GIBCO),100 U/mL penicillin and 0.1 mg/mL streptomycin (GIBCO) as previously described [26]. On day 0, the OP9 cells were plated in 24-well-plates at a density of 2 x 10^4^ cells/well. On day 1, OP9 medium was replaced by 1 mL of IMDM medium supplemented with 10% FCS, mIL3 (10 ng/mL), mSCF (30 ng/mL), mFlt3l (25 ng/mL), mGM-CSF (10 ng/mL), mIL11 (25 ng/mL), huEPO (4 U/mL) and mTPO (25 ng/mL) with or without 10 μM DAPT (γ-secretase inhibitor, Sigma) and seeded with 2000 sorted MEPs. After a 2 days culture, the non adherent MEP cells progeny was collected and remaining cells adhering to OP9 stromal cells were recovered after treatment with 0.25% trypsin at 37°C followed by an 1 hour decantation in 10 mL of IMDM medium containing 10% FCS performed in 10 cM Petri dish to remove OP9 cells. The totality of adherent and non-adherent MEP cells progeny collected in each well was then analyzed by colony assay.

### Colony assays

Colony assays were performed by duplicate seeding of 1000 or 2000 of freshly sorted MEPs and/or of the totality of their day 2 progeny into 3 mL final volume of MethoCult^R^ M3234 (StemCell Technologies) supplemented with 30% FCS, mIL3 (10 ng/mL), mSCF (50 ng/mL), mFlt3l (5 ng/mL), mGM-CSF (5 ng/mL), mIL11 (50 ng/mL), huEPO (4 U/mL) and mTPO (50 ng/mL) allowing the growth of all types of myeloid progenitors. Mixed erythro-megakaryocytic, erythroid, megakaryocytic and myeloid colonies were scored under microscope after 7 days of culture at 37°C, 5% CO_2_. All cytokines were purchased from PeproTech except huEPO (kindly provided by F Nicolini).

### Cell cycle analyses

For cell cycle analyses, sorted CD9^High^ and CD9^Med^ MEPs were centrifuged at 400 g for 10 minutes, fixed using Foxp3/Transcription Factor Staining Buffer Set (eBioscience), treated with DNAse free RNAseA (100 μg/mL for 30 min at room temperature) and labeled using FITC mouse anti-human Ki-67 Set (BD Pharmingen) and 50 μg/mL propidium iodide followed by FACS analysis. Percentages of cells in G1/G0, S and G2/M phases of cell cycle were determined using FloJo software.

### Polyploidy analyses

Polyploidy analyses were performed at day 5 of CD9^High^ MEP cultures. Cells were centrifuged at 400 g for 10 minutes, labeled with anti-c-kit-APC (BD Pharmingen), anti-CD41-FITC (eBiosciences) and anti-CD42b-PE (Emfret) antibodies. DNA was stained with Hoechst 33342 (20 μg/mL Eurogentec) during 45 min at 37°C followed by FACS analysis after gating on the Kit^-^/CD41^+^/CD42b^+^ mature megakaryocytic subset from which the percentages of cells displaying different levels of DNA content were recorded.

### Single cell liquid cultures

Sorted CD9^High^ or CD9^Med^ MEP subsets were seeded as single cell in wells of 96 wells culture plates coated with either IgGs or rDLL1 and cultured in IMDM medium supplemented as for batch cultures. Colonies were scored after 7 days under bright field microscope as described previously [37] and illustrated in S8 Fig. Myeloid cells containing colonies were further confirmed by FACS analysis of CD11b expression.

### qRT-PCR analyses

Total RNA was extracted using the Rneasy PLUS microkit (Qiagen) and reverse transcribed using a Quantitect reverse transcription kit (Qiagen). qPCR reactions were performed on a Mx3000P qPCR instrument (Stratagene) using Light-cycler 480 SybR-Green-Master-Roche kit and primers indicated in S1 Table. mRNA specific signals were normalized to that of beta-actin mRNA.

### Statistics

Quantitative data were analyzed by ANOVA followed by Tukey’s post-hoc test with the help of XLSTAT-Premium software as well as by Student t-test. Differences were considered statistically significant at p < 0.05.

## Results

### Notch activation stimulates the amplification of bipotent and erythroid progenitors derived from MEP population

**Fig 1:**
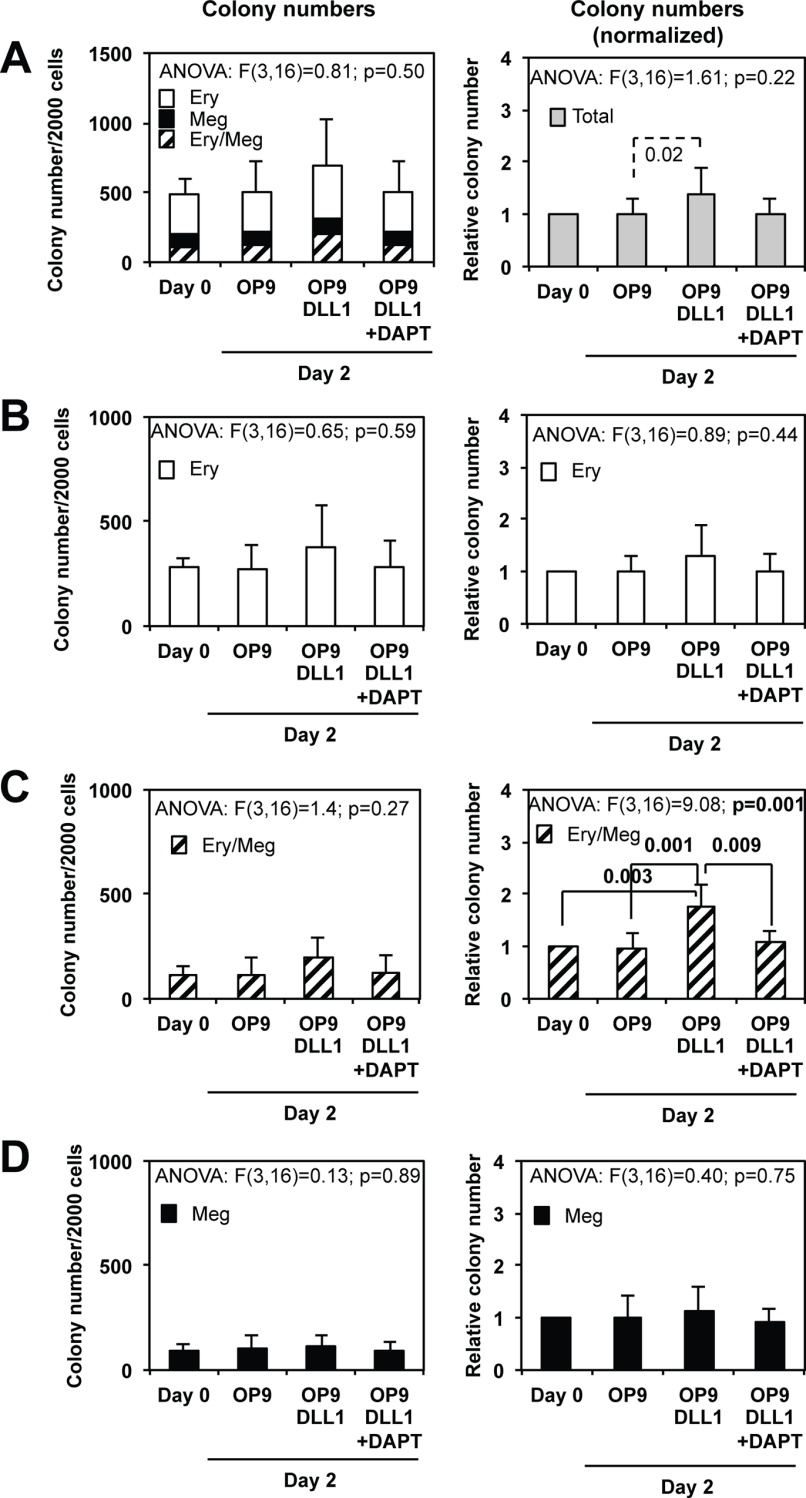
MEP cells culture on OP9 stromal cells expressing Notch ligand DLL1 stimulates the amplification of bipotent progenitors. 2000 bone marrow MEP cells were cultured for two days in the presence of a complete cocktail of myeloid cytokines (IL3, SCF, EPO, GM-CSF, TPO, Flt3L, IL11) either on control OP9 or on OP9-DLL1 stromal cells expressing Notch ligand DLL1 in the presence or absence of γ-secretase inhibitor DAPT as indicated. Total numbers of bipotent erythro-megakaryocytic (Ery/Meg), pure erythroid (Ery) or megakaryocytic (Meg) progenitors present in the initial population (day 0) and after the two days culture in the different conditions were determined by colony assays performed in semi-solid medium in the presence of the same complete cocktail of cytokines. Absolute and relative numbers of different types of colonies (normalized to that obtained on day 0) are presented on left and right histograms respectively (means and standard deviations from 5 independent MEP preparations). **A**: Left panel shows piled histograms of the numbers of erythroid (Ery), megakaryocytic (Meg) and mixed (Ery/meg) colonies generated from untreated cells (Day 0) and after a two days culture on OP9, OP9-DLL1 or OP9-DLL1 stromal cells + DAPT. Right histograms show the relative total numbers of colonies. **B, C, D:** Histograms showing separately the numbers (left panels) and relative numbers (right panels) of erythroid (**B**), mixed (**C**) and megakaryocytic (**D**) colonies obtained in the different culture conditions (same data as in **A**). Results of ANOVA analyses performed on each dataset are indicated above each corresponding histograms. Statistically significant differences between conditions are indicated by braces with corresponding post-hoc p-values for the Tukey’s test indicated in bold characters. Statistically significant differences validated in Student t-test only are indicated by dotted braces.

The first aim of our study was to determine the fate of erythro-megakaryocytic bipotent progenitors upon stimulation of the Notch pathway. For that purpose, we used colony assays to quantify the number of different progenitors present in sorted mouse bone marrow MEP population (lin^-^/Sca1^-^/Kit^+^/CD16/32^low^/CD34^low^; S1 Fig) before and after a 2 days stimulation of the Notch pathway in the presence of a complete cocktail of myeloid cytokines (IL3, Ftl3l, GM-CSF, SCF, IL11, EPO, TPO). 25% of freshly sorted MEPs generated colonies including 50% of pure erythroid colonies, 25% of pure megakaryocytic and 25% of mixed erythro-megakaryocytic colonies (Fig 1A, Day 0). These results confirmed previous studies, that the MEP population is actually composed of a mixture of pure erythroid and megakaryocytic progenitors in addition to bipotent progenitors. We also confirmed that this MEP population expressed Notch2 receptor (FACS analysis not shown) as previously reported by others [36].

In a first series of experiments, sorted MEPs were co-cultured for 2 days either with control OP9 stromal cells or with OP9-DLL1 cells expressing DLL1 Notch ligand, and in the presence or absence of γ-secretase inhibitor DAPT. When compared with Day 0, co-cultures of MEPs with OP9-DLL1 cells slightly increased the total number of colonies (Fig 1A) and significantly increased the number of mixed colonies (Fig 1C). Moreover, these changes were reversed in the presence of DAPT and were not observed with OP9 cells attesting for a specific effect of Notch pathway activation. In the same conditions, the number of megakaryocytic colonies did not change significantly (Fig 1D) whereas a slight, but not significant, increase in erythroid colonies was observed in OP9-DLL1 co-cultures (Fig 1B).

**Fig 2:**
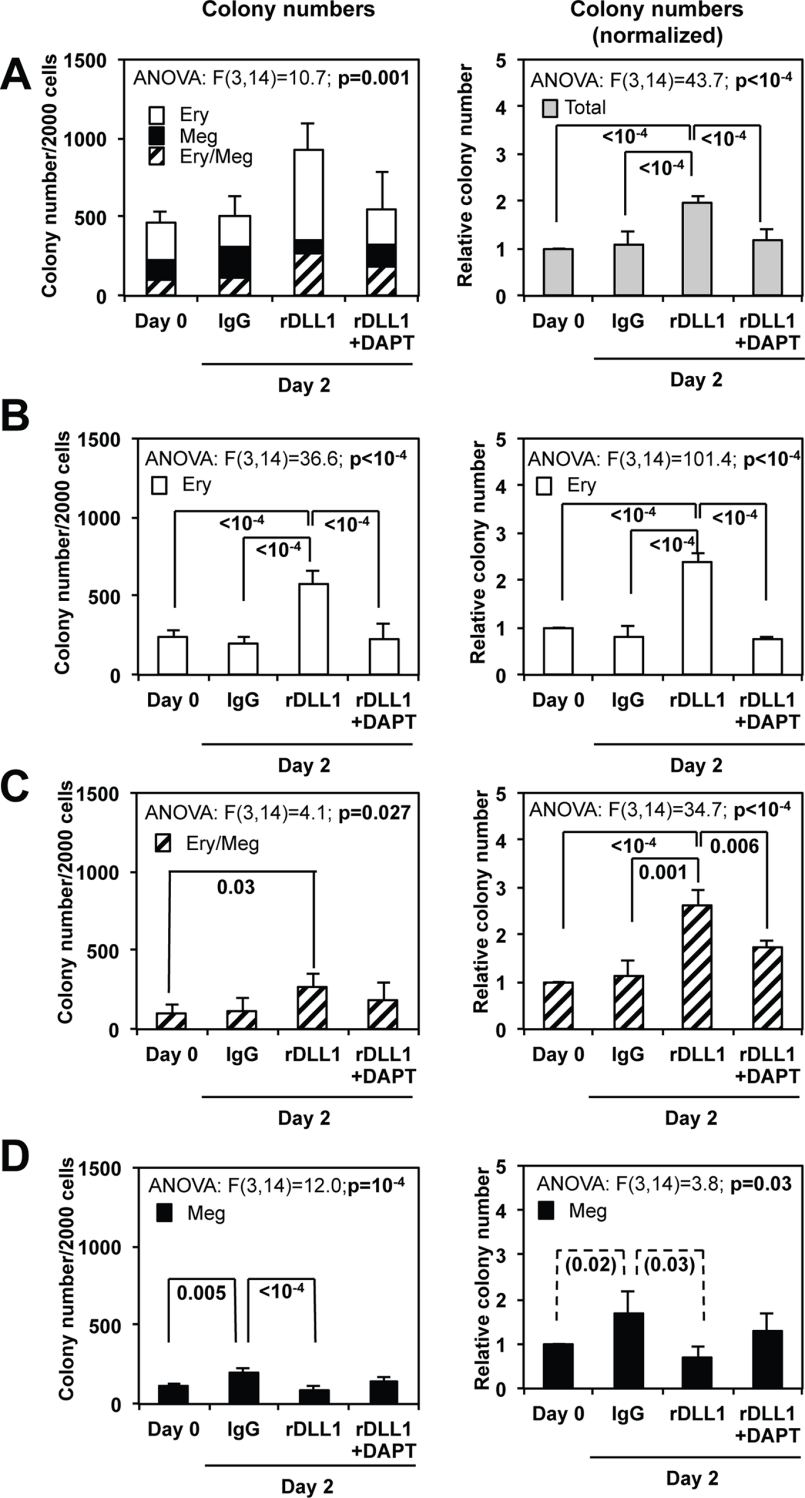
MEP cells culture on recombinant Notch ligand rDLL1 stimulates the amplification of bipotent and erythroid progenitors. 2000 bone marrow MEP cells were cultured for two days in the presence of a complete cocktail of myeloid cytokines (IL3, SCF, EPO, GM-CSF, TPO, Flt3L, IL11) in plate culture wells coated with either control IgG1 or recombinant rDLL1 in the presence or absence of DAPT as indicated. Total numbers of the bipotent erythro-megakaryocytic (Ery/Meg), pure erythroid (Ery) or megakaryocytic (Meg) progenitors present in the initial population (day 0) and after the two days culture in the different conditions were determined by colony assays performed in semi-solid medium in the presence of the same complete cocktail of cytokines. Absolute and relative numbers of different types of colonies (normalized to that obtained on day 0) are presented on left and right histograms respectively (means and standard deviations from 5 independent MEP preparations). **A**: Left panel shows piled histograms of the different types of colonies generated from untreated cells (Day 0) and after a two days culture on IgG, rDLL1 or rDLL1 + DAPT. Right histograms shows the relative total numbers of colonies. **B, C, D**: Histograms showing separately the numbers (left panels) and relative numbers (right panels) of erythroid (**B**), mixed (**C**) and megakaryocytic (**D**) colonies obtained in the different culture conditions (same data as in **A**). Results of ANOVA analyses performed on each dataset are indicated above each corresponding histogram. Statistically significant differences between conditions are indicated by braces with corresponding post-hoc p-values for the Tukey’s test indicated in bold characters. Statistically significant differences validated in Student t-test only are indicated by dotted braces.

In a second series of experiments, sorted MEPs were cultured for 2 days either on coated IgGs or on coated recombinant rDLL1 Notch ligand in the presence or absence of γ-secretase inhibitor DAPT. Again, when compared with Day 0, the 2 days culture of MEPs on rDLL1 significantly increased the total number of colonies (Fig 2A) as well as the number of mixed colonies (Fig 2C) while these changes were reversed by DAPT and were not observed after culture on control IgGs. In the same conditions but in contrast to co-cultures with OP9-DLL1 cells, cultures on rDLL1 significantly decreased the number of megakaryocytic colonies (Fig 2D) and significantly increased the number of erythroid colonies (Fig 2B). Interestingly, the net increase in erythroid colonies (mean increase of 350 colonies; Fig 2B left panel) largely exceeded the small decrease in megakaryocytic colonies (mean decrease of 50 colonies; Fig 2D left panel) thus suggesting the selective amplification of committed erythroid progenitors. Importantly, all changes observed on rDLL1 were reversed by DAPT and were not observed in cultures performed on IgGs. Moreover, qRT-PCR analyses performed at day 2 confirmed the expected increase in Hes1 transcripts consistent with Notch pathway activation in the presence of rDLL1 but not on IgGs and its partial repression by DAPT (S2 Fig). In complementary experiments, we determined that the increase of mixed and erythroid colonies induced by rDLL1 remained strictly dependent on the presence of SCF (S3 Fig) and associated with increased expression of c-Kit (S2 Fig).

Altogether, these results indicated that activation of the Notch pathway stimulates the SCF-dependent amplification of bipotent as well as erythroid progenitors derived from sorted MEPs.

### Notch activation restores erythroid and mixed potential of a CD9^High^ MEP subset committed towards megakaryocytic lineage

**Fig 3:**
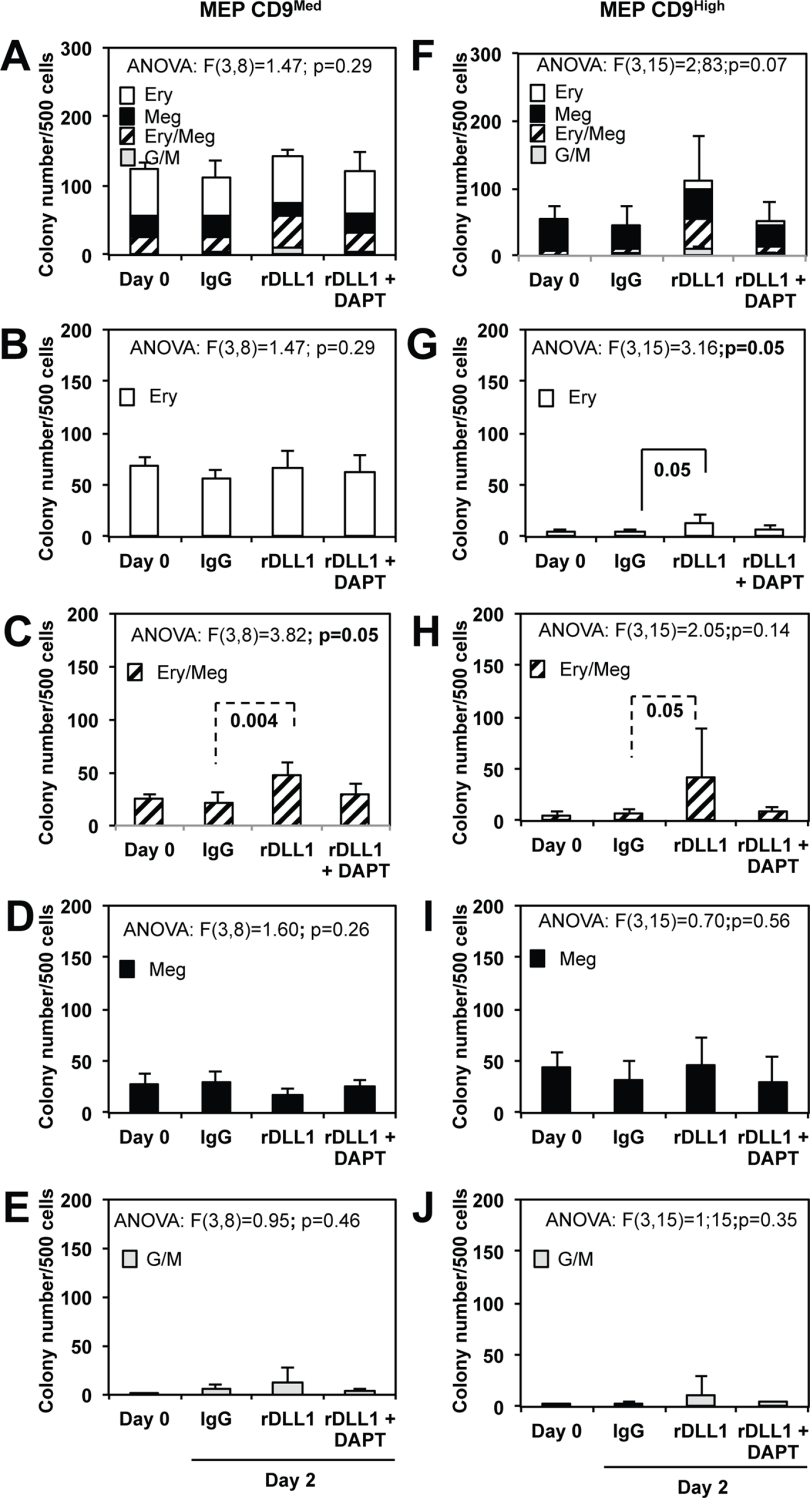
Comparison of the Notch response of sorted MEP cells expressing different levels of CD9. Progeny analyses of equal numbers of sorted CD9^Med^ and CD9^High^ MEP subsets were performed by colony assays before (Day 0) and after a two days culture either on IgG or rDLL1 with or without DAPT as described in Fig 2. The numbers of different types of colonies generated by either CD9^Med^ or CD9^High^ MEP are presented on left (A, B, C, D, E) and right (F, G, H, I, J) histograms respectively (means and standard deviations from 3 independent MEP preparations). Results of ANOVA analyses performed on each dataset are indicated above each corresponding histogram. **A, B:** Piled histograms showing the cumulated numbers of the different types of colonies generated by CD9^Med^ (A) or CD9^High^ MEP (F). Results of ANOVA analysis performed on the total numbers of colonies are indicated above the histogram. **B, G:** Histograms showing the numbers of erythroid colonies generated by CD9^Med^ (B) or CD9^High^ MEP (G). **C, H:** Histograms showing the numbers of mixed colonies generated by CD9^Med^ (C) or CD9^High^ MEP (H). **D, I:** Histograms showing the numbers of megakaryocytic colonies generated by CD9^Med^ (D) or CD9^High^ MEP (I). **E, J:** Histograms showing the numbers of myeloid colonies generated by CD9^Med^ (E) or CD9^High^ MEP (J). Statistically significant differences between conditions validated by either ANOVA analyses followed by Tukey’s test or by Student t-test are indicated by full and dotted braces respectively associated with corresponding p values.

The next step of our study was to reinvestigate the controversial effect of Notch pathway on megakaryocytic differentiation using purified megakaryocytic progenitors that are present in the MEP population. Based on several recent studies [40, 41], we reasoned that committed megakaryocytic progenitors present in the MEP population should be purified based on their high level of CD9 expression. As shown in S4 Fig, MEP cells displayed a roughly bimodal distribution of CD9 expression level allowing the sorting of three different subsets according to their CD9 expression level (CD9^Low^, CD9^Med^ and CD9^High^). Colony assays showed that the proportion of megakaryocytic colonies positively correlated with the expression level of CD9 reaching more than 90% of megakaryocytic colonies generated by CD9^High^ subset in association with a reduced clonogenicity (S4 Fig). Since the CD9^Low^ subset appeared to be slightly contaminated by a few proportion of GMPs (S4 Fig), we focused our next analyses on the comparison of the Notch response between CD9^Med^ and CD9^High^ subsets that contained most of the erythroid-megakaryocytic potential with minimal GMP contamination. For that purpose, we followed the same protocol described in Fig 2 and the results obtained with three independent preparations of CD9^Med^ or CD9^High^ cells are presented in Fig 3A–3E and 3F–3J respectively. As expected, CD9^Med^ MEPs (representing about 50% of total MEP cells), roughly reproduced results obtained with the unfractionated MEP population notably the significant increase of bipotent colonies (Fig 3C) together with a slight but not significant decrease of megakaryocytic colonies after the 2 days culture on rDLL1 instead of IgG (Fig 3D). In contrast, the very low numbers of erythroid and bipotent colonies generated by CD9^High^ MEPs at day 0 or after the 2 days culture on IgG were markedly enhanced (up to 10 and 40 fold respectively) after the 2 days culture on rDLL1 (Fig 3G and 3H). These results thus indicated that Notch activation unexpectedly induced the generation of mixed colonies by CD9^High^ MEPs that otherwise spontaneously generate only megakaryocytic colonies.

### Notch activation increases the size of megakaryocytic colonies and decreases the maturation and polyploidy of megakaryocytes generated by CD9^High^ MEPs

**Fig 4:**
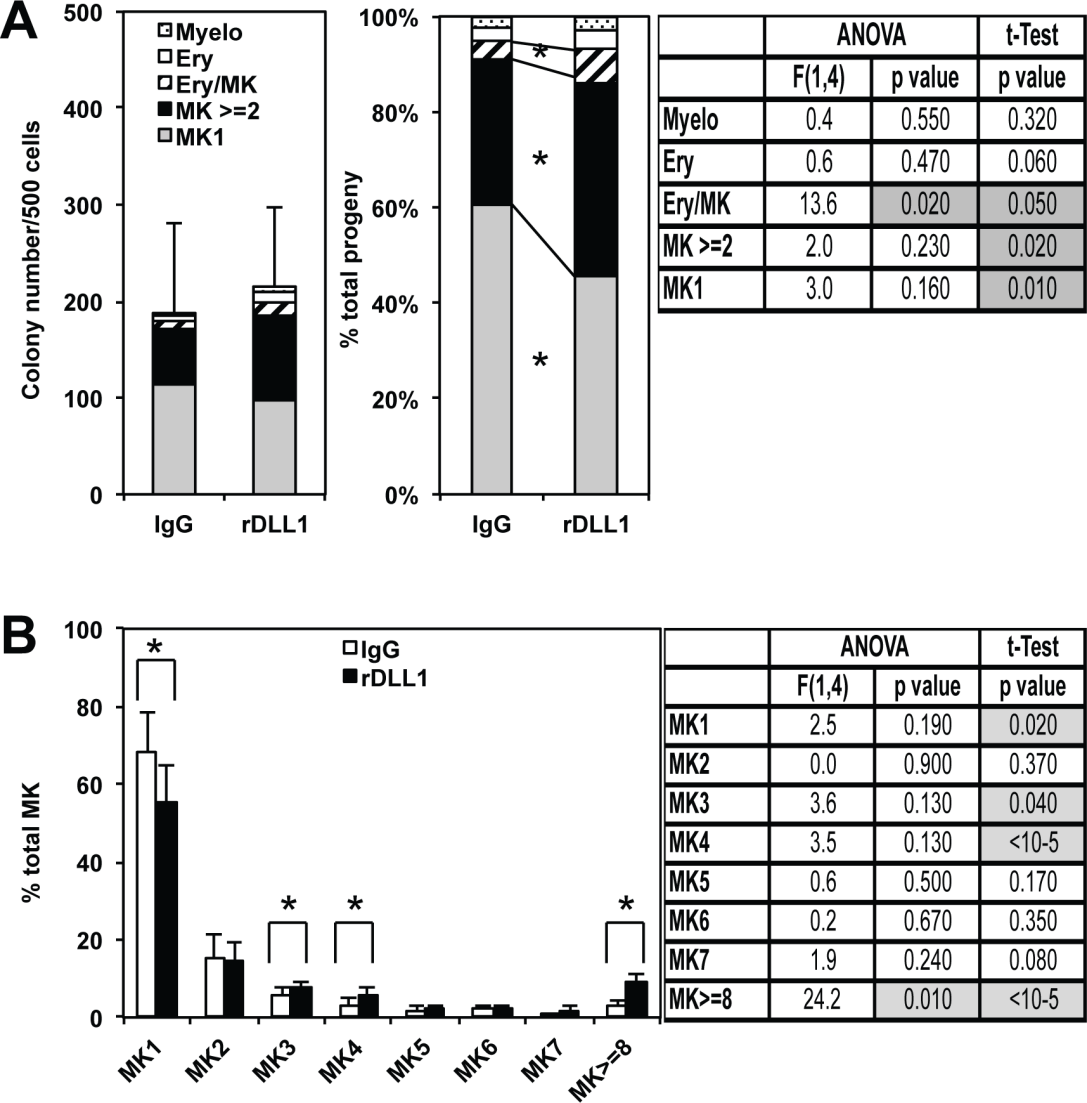
Culture on rDLL1 decreases the number of single megakaryocytes and increases the size of megakaryocytic colonies generated by CD9^High^ MEPs. CD9^High^ MEPs were cultured for two days on either IgGs or rDLL1 before analysis of their progeny by colony assays as described in Fig 3 except that single megakaryocytes and megakaryocytic colonies displaying different numbers of megakaryocytes were numbered separately. **A:** Piled histograms showing the numbers (left panel) and percentages (right panel) of single megakaryocytes (MK1), megakaryocytic colonies containing at least 2 megakaryocytes (MK ≥2) as well as few erythroid (Ery), myeloid (Myelo) and mixed erythro-megakaryocytic (Ery/Mk) colonies generated after the 2 days culture on either IgGs or rDLL1 (mean and standard deviations from 3 independent MEP preparations). Table on the right displays p-values in Tukey’s test post ANOVA and in Student t-test analyses of the variations in the proportions of different types of colonies between IgG and rDLL1 conditions. Statistically significant variations are indicated by grey boxes in table and by asterisks on the right histogram. **B:** Histograms showing the percentages of different sizes of pure megakaryocytic colonies (from single to 8 and more megakaryocytes) on IgGs or rDLL1 (means and standard deviations from the same 3 independent MEP preparations as in A). Table on the right displays p-values in Tukey’s test post ANOVA and in Student t-test analyses of the variations in the proportions of different types of colonies between IgG and rDLL1 conditions. Statistically significant variations are indicated by grey boxes in Table and by full braces and asterisks on histogram.

Before investigating the origin of the increase of erythroid and bipotent colonies by CD9^High^ MEPs, we decided to characterize the effect of rDLL1 on the size and maturation of pure megakaryocytic colonies. We noticed that up to 90% of the viable progeny generated by CD9^High^ MEPs after a 2 days culture on IgGs were actually exclusively composed of megakaryocytic colonies and up to 60% of single megakaryocytes (Fig 4A). While the total number of this viable progeny did not significantly change on rDLL1 culture (Fig 4A, left panel), the proportion of single megakaryocytes significantly decreased for the benefit of a significantly increased proportion of megakaryocytic and bipotent colonies (Fig 4A, right panel). Moreover, by counting the relative proportions of megakaryocytic colonies with various numbers of megakaryocytes, we found a slight (-10%) but significant decrease in single megakaryocytes (Fig 4B, MK1) and a significant increase in colonies displaying 3, 4 or more than 8 megakaryocytes generated by CD9^High^ MEPs cultured on rDLL1 versus IgGs (Fig 4B). Importantly, this increase in the size of megakaryocytic colonies as well as the increase in bipotent colonies induced by rDLL1 were abrogated in the presence of either DAPT or Notch2 neutralizing antibody thus indicating that these effects are specifically induced by Notch2 receptor activation (S5 Fig). These results thus indicated that most (>90%) of CD9^High^ MEPs were indeed strongly biased towards megakaryocytic differentiation and that their culture on rDLL1 induced them to undertake additional divisions before terminal differentiation.

**Fig 5:**
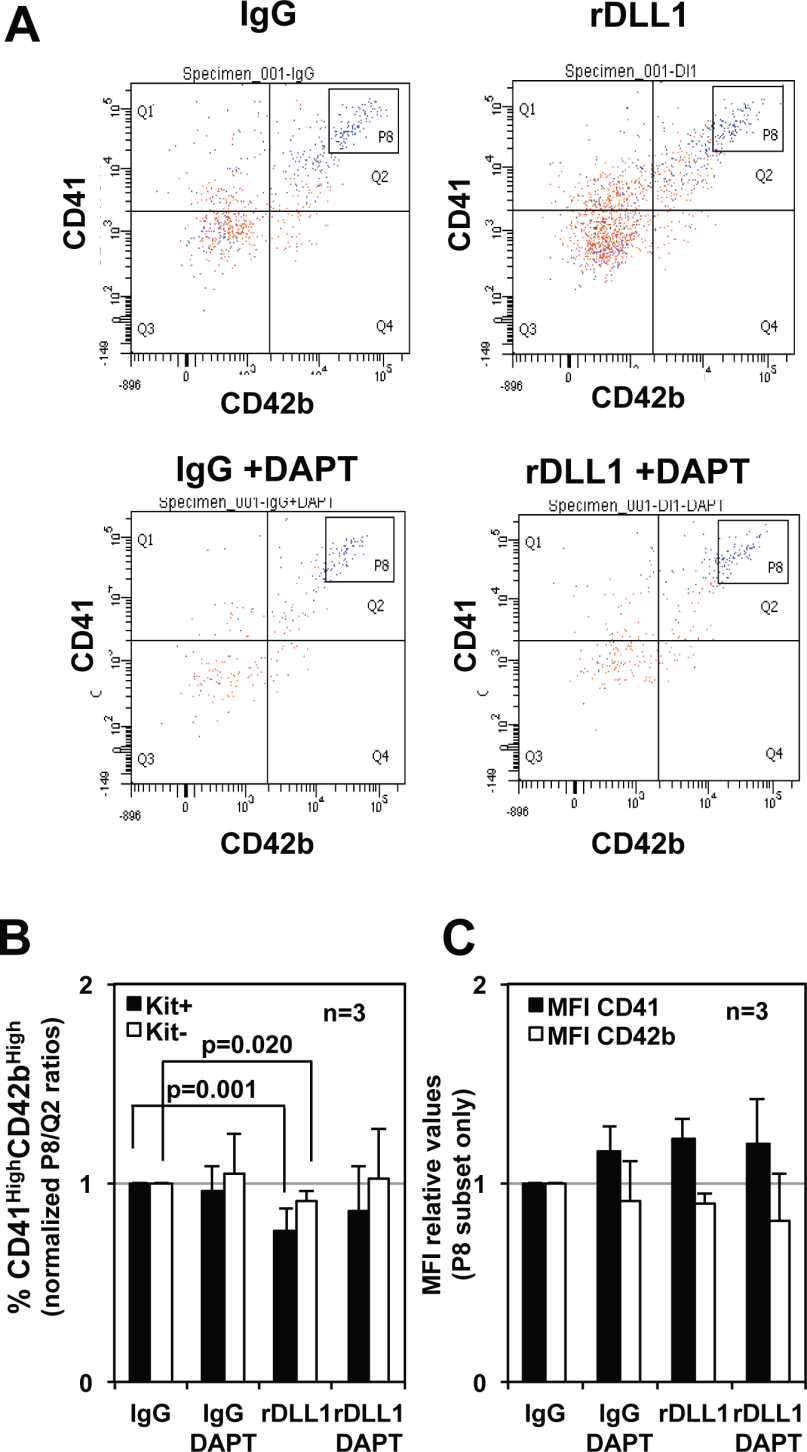
Culture on rDLL1 slightly delays the maturation of megakaryocytes generated by CD9^High^ MEPs. Equal numbers of CD9^High^ MEP cells were cultured for 5 days in the presence of a complete cocktail of myeloid cytokines (IL3, SCF, EPO, GM-CSF, TPO, Flt3L, IL11) in plate culture wells coated with either control IgG1 or recombinant rDLL1 in the presence or absence of DAPT followed by FACS analyses after labeling with cKit, CD41 and CD42b fluorescent antibodies. **A:** FACS dot-plots showing the expression of CD41 and CD42b with red and blue dots corresponding to cKit^+^ and cKit^-^ cells respectively. Gate P8 is defined by CD41^+^CD42b^+^ double positive cells displaying the highest levels of both CD41 and CD42b (CD41^High^CD42b^High^). Note that most cells in gate 8 do not express c-Kit supporting our interpretation that they correspond to the most mature megakaryocytes. **B:** Histograms showing relative proportions of most mature CD41^High^CD42b^High^ megakaryocytes (gate 8) among all CD41^+^CD42b^+^ cells (gate Q2) including (black bars) or not (white bars) Kit^+^ cells (Means and standard deviations from 3 independent experiments). Statistically significant variations are indicated by braces with corresponding p-values in Student t-test. **C:** Histograms showing the absence of significant variations in the relative MFIs of CD41 (black bars) and CD42b (white bars) among CD41^High^CD42b^High^ cells.

In a complementary approach, we used flow cytometry analyses to compare the maturation and polyploidization of megakaryocytes generated by CD9^High^ MEPs after a 5 days culture on either IgGs or rDLL1. Semi-quantitative analyses revealed that CD9^High^ MEPs generated an increased number of viable cells (mainly Kit^+^ progenitor cells) on rDLL1 than on IgGs (S6 Fig) but the proportion of maturing CD41^+^/CD42b^+^ double positive megakaryocytic cells did not change significantly (data not shown). However, we found that the culture on rDLL1 induced a slight but significant (p = 0.01 in Student t-test) decrease in the proportion of CD41^+^/CD42b^+^ cells displaying the highest levels of both CD41 and CD42b (CD41^High^CD42b^High^ gate 8, Fig 5A and 5B) when compared to other control conditions. Interestingly, the decrease in the proportion of these CD41^High^CD42b^High^ megakaryocytes (most of which did not express c-kit; Fig 5A) was attenuated among Kit^-^CD41^+^CD42b^+^ (Fig 5B; compare black and white bars) and their mean expression levels of CD41 and CD42b (Fig 5C) did not change significantly thus suggesting a delay rather than strong inhibition of terminal megakaryocytic differentiation.

**Fig 6:**
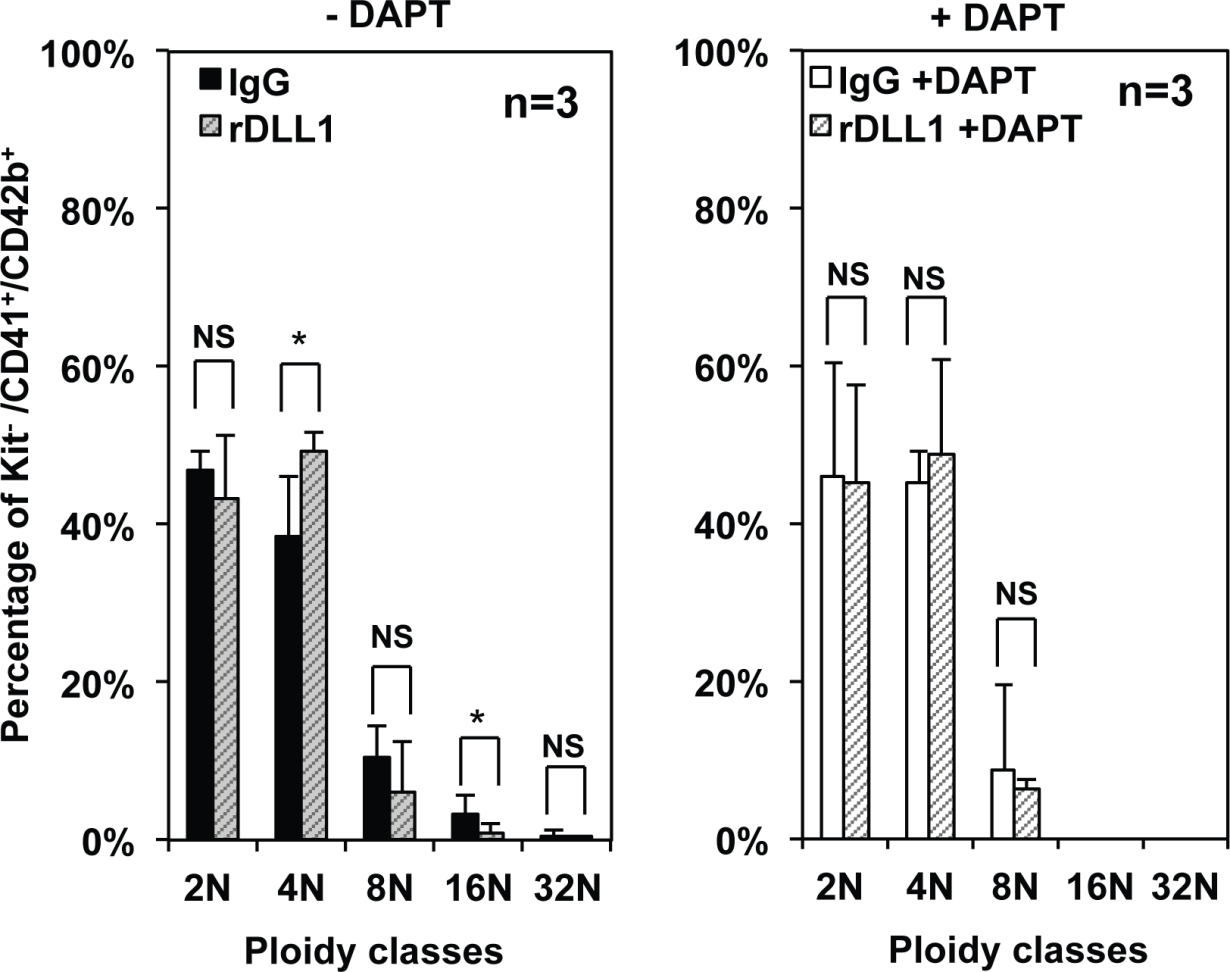
Culture on rDLL1 decreases the ploidy of megakaryocytes generated by CD9^High^ MEPs. Equal numbers of CD9^High^ MEP cells were cultured for 5 days in the presence of a complete cocktail of myeloid cytokines (IL3, SCF, EPO, GM-CSF, TPO, Flt3L, IL11) in plate culture wells coated with either control IgG1 or recombinant rDLL1 in the presence or absence of DAPT followed by FACS analyses after labeling for CD41, CD42b, c-Kit and DNA content. Histograms show the percentages of the different classes of polyploidy of Kit^-^/CD41^+^/CD42b^+^ cells generated on IgGs (full boxes) or rDLL1 (hatched boxes) in the absence (left panel) or presence (right panel) of DAPT (Means and standard deviations from 3 independent experiments). Statistically significant variations between conditions (p < 0.05 in Student t-test) are indicated by asterisks (NS: non significant variation).

Polyploidization of megakaryocytes generated in the same culture conditions was addressed by FACS analyses after DNA labeling. We found a 2 fold decrease in the mean percentage of polyploid Kit^-^/CD41^+^/CD42b^+^ cells generated on rDLL1 as compared with IgGs (7 ± 6% of ≥ 8N on rDLL1 vs 15 ± 5% on IgGs) with a significant decrease in the percentage of 16 N cells (Fig 6A). However, the percentage of 4N Kit^-^/CD41^+^/CD42b^+^ cells (including cells in S phase) generated on rDLL1 significantly increased as compared with IgGs (Fig 6A) thus suggesting concomitant increase of cycling cells as also suggested by cell cycle analyses performed at day 2 (S7 Fig). Importantly, none of these changes were detected in the presence of DAPT (Fig 6B).

Altogether, these data thus suggested that MK-committed progenitors, constituting more than 90% of CD9^High^ MEPs, are induced to perform additional divisions before terminal megakaryocytic differentiation and polyploidization upon Notch stimulation.

### Notch activation favors the commitment of CD9^Med^ MEPs towards erythroid at the expense of megakaryocytic lineages

**Fig 7:**
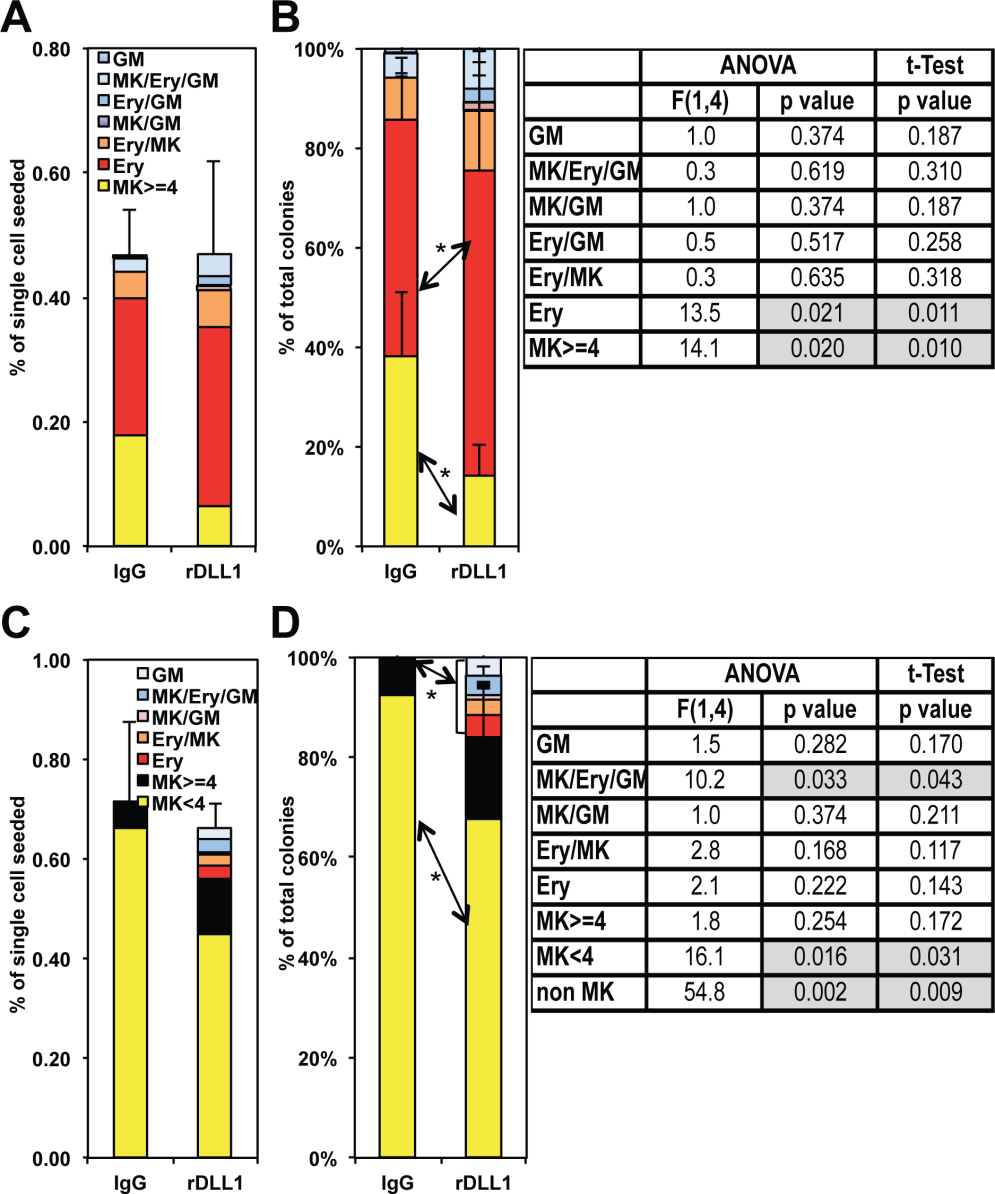
Single cell progeny analyses of CD9^Med^ and CD9^High^ MEPs with or without Notch activation. Single CD9^Med^ or CD9^High^ MEP were individually seeded in 96 wells culture plates that have been coated with either IgGs or rDLL1 and containing medium supplemented with a complete cocktail of myeloid cytokines. The different types of developed colonies were numbered at day 7 by visual inspection under bright light microscope as illustrated in S8 Fig. **A, B**: Repartition of the indicated type of colony as a percentage of all single seeded CD9^Med^ MEP (**A**) and as a percentage of CD9^Med^ MEP raising colonies (**B**) (Means and standard deviations from 3 independent experiments). **C, D**: Repartition of the indicated type of colony as a percentage of all single seeded CD9^High^ MEP (**C**) and as a percentage of CD9^High^ MEP raising colonies (**D**) (Means and standard deviations from 3 independent experiments). Tables on the right display p-values in Tukey’s test post ANOVA and in Student t-test analyses of the variations in the proportions of different types of colonies between IgG and rDLL1 conditions. Statistically significant variations are indicated by grey boxes in Tables and by asterisks on right histograms.

Based on the above results (Figs 2 and 3), the question remained of whether, in addition to its proliferative effect on committed erythroid progenitors, Notch activation could also favor the preferential commitment of bipotent progenitors towards the erythroid lineage. To directly address this question, single CD9^Med^ MEP cells were individually seeded in wells coated with either IgGs or rDLL1 and their progeny was characterized after a 9 days culture by morphological examination as illustrated in S8 Fig. As expected, single CD9^Med^ MEPs generated colonies containing either only large megakaryocytic cells, only small erythroid cells, or both erythroid and megakaryocytic cells (Fig 7A). A few number of mixed colonies also contained granulo-monocytic cells identified by their intermediate size and expression of CD11b (Fig 7A). While the proportion of single CD9^Med^ MEPs generating colonies did not significantly change when cultured on either IgGs or rDLL1 (Fig 7A), the proportion of CD9^Med^ MEPs that led to erythroid colonies increased at the expense of those giving rise to megakaryocytic colonies (only colonies containing over 4 cells were scored) in the presence of rDLL1 as compared with IgGs (Fig 7B). These results thus indicated that Notch activation indeed favored the commitment of CD9^Med^ MEP towards erythroid lineage at the expense of megakaryocytic lineage.

### Notch activation restores erythroid and mixed potential in a fraction of CD9^High^ MEPs

The above results also raised the question of whether the striking restoration of erythroid and mixed colonies by CD9^High^ MEPs in response to Notch ligand (Figs 3F and 4A) was due to a real change of their megakaryocytic fate and/or to the reactivation of few contaminating multipotent and quiescent progenitors. We noticed that CD9^High^ MEPs were actually characterized by the presence of cells with 4N DNA content and reduced Ki67 expression levels (S7 Fig, arrow) as well as 5% of binucleated cells (S7 Fig) that may correspond to megakaryocytes transiently paused during the process of their polyploidization [42, 43]. In contrast, we found that CD9^High^ MEPs harbored a very low proportion (less than 5%) of quiescent G0 cells (2N/Ki67^neg^) whereas G0 accounted for up to 30% of CD9^Med^ MEPs (S7 Fig). These observations prompted us to address directly whether Notch activation could be able to change the megakaryocytic fate of CD9^High^ MEPs by performing single cell progeny analyses. Up to 70% of single CD9^High^ MEPs cultured on IgGs generated a viable and exclusively megakaryocytic progeny (Fig 7C) including 90% of colonies containing less than 4 megakaryocytes (Fig 7D). The proportion of single CD9^High^ MEPs generating a viable progeny did not change significantly when cultured on either IgGs or rDLL. Moreover, CD9^High^ MEPs displayed a similar proportion of DAPI^neg^ viable cells after a 2 days culture on either IgGs or rDLL1 (61.9% DAPI^neg^ on IgG vs 58.5% DAPI^neg^ on rDLL1) thus further indicating that Notch activation did not significantly impact CD9^High^ MEPs survival. Remarkably, whereas the progeny of single CD9^High^ MEPs on IgGs was exclusively composed of megakaryocytic cells, up to 15% of this progeny was committed into either erythroid, erythro-megakaryocytic or even mixed colonies containing granulo-monocytic cells on rDLL1 culture (Fig 7D). Moreover, confirming results obtained by colony assays performed in semi-solid medium (Fig 4B), the proportion of small colonies containing less than 4 megakaryocytes significantly decreased on rDLL1 as compared with IgGs. Altogether, these single cell progeny analyses thus indicated that the restoration of erythroid and mixed colonies induced by Notch occurred mainly at the expense of megakaryocytic colonies thus strongly suggesting a commitment effect rather than a simple reactivation of quiescent cells.

### The two days culture of CD9^High^ MEP increases Klf1 and Aurka gene transcripts levels

In spite of the low proportion of CD9^High^ MEPs changing their fate upon Notch activation, we tried to determine whether these changes could be associated with significant changes in the expression of few important regulators involved in the control of erythroid versus megakaryocytic commitment and of terminal megakaryocytic differentiation. In addition to the expected increase of Hes1 transcripts, we found a slight increase of transcripts encoding KLF1 transcription factor (S9 Fig) already known to favor erythroid at the expense of megakaryocytic lineage commitment [44]. Interestingly enough, we also found increased levels of transcripts encoding Aurora A kinase (S9 Fig) recently identified as a cell cycle regulator required for proliferation and survival of all hematopoietic progenitors but which must be down regulated to allow terminal megakaryocytic maturation and polyploidization [45]. Importantly, all these changes in transcript levels were not observed in the presence of DAPT. These findings thus indicate that Notch activation modulates the expression of at least two major regulators controlling erythroid versus megakaryocytic commitment and differentiation in a way that could explain the re-expression of erythroid potential and the decrease in terminal megakaryocytic differentiation of CD9^High^ MEPs.

## Discussion

The aim of this study was to investigate the effect of the Notch pathway on the development of the megakaryocytic lineage. To specifically answer this question, we combined colony assays as well as bulk and single cell progeny analyses performed on pure erythro-megakaryocytic bipotent MEP populations before and after short-term cultures in the presence or absence of recombinant Notch ligand rDLL1. This strategy allowed us to distinguish between the effects of Notch on the commitment of bipotent progenitors from its differential effects on the proliferation of uncommitted and/or fully committed progenitors.

Our colony assays first confirmed the heterogeneity of freshly sorted MEP populations actually composed of a mixture of truly bipotent and pure erythroid or megakaryocytic progenitors. Progeny analyses of whole MEP populations showed that activation of the Notch pathway (evidenced by increased levels of the Notch target Hes1 mRNA) stimulates the SCF-dependent amplification of progenitors in short-term cultures performed on rDLL1. Colony assays further showed that amplified progenitors were mainly bipotent and erythroid progenitors. Moreover, the increase in the number of erythroid progenitors largely exceeded the small decrease in the number of megakaryocytic progenitors thus further suggesting the selective amplification of committed erythroid progenitors (Fig 2). Finally, single cell progeny analyses directly showed that Notch favored the commitment of bipotent progenitors towards the erythroid lineage at the expense of the megakaryocytic lineage (Fig 7A). Altogether, these results indicate that Notch modulates the production of erythroid and megakaryocytic progenitors by at least two ways: (i) by favoring the commitment of bipotent progenitors towards the erythroid lineage and (ii) by stimulating the amplification of bipotent as well as already committed erythroid progenitors. This conclusion corroborates two recent studies showing the contribution of Notch to the SCF-dependent amplification of human erythroid progenitors *in vitro* [35] as well as murine erythroid progenitors during stress erythropoiesis *in vivo* [36]. This conclusion further strengthens the role of Notch in favoring erythroid commitment as previously suggested by the increased proportion of erythroid colonies generated by murine PreMegE bipotent progenitors expressing Hes1 [36]. Our present demonstration that Notch can stimulate the amplification of bipotent progenitors is a new finding that also helps to understand some of the controversy regarding the role of Notch as an activator of megakaryocytic development. Indeed, this finding provides an alternative interpretation for previous experiments showing increased production of MEP and CD41^+^ progenitors upon Notch pathway activation that led to the conclusion that Notch favors megakaryocytic lineage [26, 27].

In addition, our study evidences the unexpected plasticity of committed megakaryocytic progenitors revealed upon Notch activation. In agreement with previous studies [40, 41], we identified a specific subset of MEPs expressing high levels of CD9 that are strongly biased towards megakaryocytic differentiation. Indeed, this CD9^High^ subset generated more that 90% of pure megakaryocytic progeny among which 60% were single megakaryocytes (Figs 3 and 4 and S5 Fig). When cultured on rDLL1 instead of IgGs, these CD9^High^ MEPs generated significantly larger megakaryocytic colonies clearly indicating the stimulation of additional divisions before terminal megakaryocytic differentiation (Figs 4 and 7A and S5 Fig). Moreover, megakaryocytes generated on rDLL1 displayed a slightly reduced polyploidy (Fig 6) and extent of maturation as appreciated by their expression levels of CD41 and CD42b (Fig 5). However, rDLL1 did not reduce maximal expression levels of CD41 and CD42b among CD41^+^CD42b^+^ckit^-^ cells thus suggesting delayed rather than strong inhibition of maturation. Likewise, rDLL1 did not reduce maximal polyploidy levels but concomitantly increased the proportion of 4N (including cells in S phase) thus suggesting delayed rather than strong inhibition of polyploidization. Importantly, all these effects induced by rDLL1 on the progeny of CD9^High^ MEPs are fully suppressed in the presence of Notch2 neutralizing antibody (S5 Fig) thus strongly supporting specific effects mediated by the activation of Notch2 receptor in agreement with its recent identification as the major Notch receptor expressed in MEP [36]. Taken together, these results support our interpretation that Notch2 activation extends the proliferative potential of MK-committed progenitors thus delaying their terminal maturation and polyploidization. Intriguingly, we noticed that the CD9^High^ subset included 5% of binucleated cells (S7 Fig) that may correspond to megakaryocytes transiently arrested in the process of their polyploidization [42, 43]. It is therefore tempting to speculate that some of these binucleated megakaryocytes might be induced to resume division instead of polyploidization in response to Notch activation.

Our most surprising observation is the striking restoration of erythroid, bipotent and even few mixed colonies containing myeloid cells by CD9^High^ MEPs upon stimulation of Notch (Figs 3, 4 and 7B and S5 Fig). Several arguments strongly argue against the simple reactivation of cell cycle in contaminating quiescent multipotent progenitors. First, we found only low proportion of quiescent G0 cells in the CD9^High^ subset while such G0 cells were readily detected in the CD9^Med^ subset (S7 Fig). Most importantly, in the three independent experiments performed totalizing the analysis of the progeny of 252 CD9^High^ single seeded cells (Fig 7B), every cell generated exclusively megakaryocytic progeny on IgGs whereas up to 15% generated progeny containing alternative lineage on rDLL1. Moreover, the generation of these 15% progeny containing alternative lineage occurred without any significant change in cell viability as appreciated by the percentage of single cells generating a viable progeny (77% ± 8 *vs* 71% ± 6 on IgGs *vs* rDLL1 respectively) or by the percentage of DAPI^neg^ viable cells detected after two days of culture (61.9% DAPI^neg^ on IgG vs 58.5% DAPI^neg^ on rDLL1). These results support our conclusion that around 15% of CD9^High^ MEPs that spontaneously generate exclusively megakaryocytic progeny are actually able to generate alternative lineage upon stimulation of Notch pathway. This interpretation is consistent with a retrospective analysis of single cell MEP transcriptome showing only slight decrease of several erythroid specific genes (KLF1, TFRC, KEL) in single CD9^+^ MEP as compared with CD9^-^ MEP (S10 Fig)[46]. We also found that the 2 days culture of CD9^High^ MEPs on rDLL1 was sufficient to induce detectable changes in the expression of two major regulators in a way that could explain the re-expression of erythroid potential and the decrease in terminal megakaryocytic differentiation. Notably, the increase in Klf1 expression is fully consistent with its known implication in erythroid at the expense of megakaryocytic lineage commitment [44]. Likewise, the increase in Aurka expression is also fully consistent with its known implication in the proliferation and survival of all hematopoietic progenitors and the requirement of its downregulation to allow terminal megakaryocytic differentiation and polyploidization [45]. Interestingly enough, similar changes in the expression of these 2 regulators are clearly detected in previous data comparing transcriptome of mouse bone marrow progenitors expressing or not Hes1 (S11 Fig) thus indicating robust regulation of these two regulators by the Notch pathway. Further studies will be required to identify the detailed molecular mechanisms of this regulation as well as to identify other Notch targets controlling the fate of MK-committed progenitors. For example, our original finding that Notch activation is able to favor the proliferation of MK-committed progenitors at the expense of their terminal differentiation is, in some way, reminiscent of the effect induced by lowering JAK2 activity that has been recently shown to change the response of megakaryocytes to thrombopoietin (TPO) from terminal differentiation to resume cell proliferation [47]. This raises in turn the intriguing possibility that Notch pathway could also contribute to maintain bi/multipotency of erythro-megakaryocytic progenitors through the control of the Jak/stat pathway possibly by maintaining oscillating activity as reported in neuronal stem cells [48, 49].

Several recent studies provided strong support for the existence of an alternative pathway bypassing classical MEPs and leading to the short cut production of fully committed megakaryocytic progenitors directly from multipotent HSCs and/or CMPs [50–52] as well as the existence of megakaryocytic-lineage restricted cells displaying self-renewal *in vivo* [53, 54]. Notably, very recent transcriptome analyses of single CD41^+^ LT-HSCs revealed the existence of a full continuum of stem cells displaying the expression an increasing number of megakaryocytic genes correlating with increasing levels of CD41 [52]. It would be therefore very interesting to know whether MK-committed progenitors directly generated by these multipotent populations are also able to extend their proliferative potential and to reactivate alternative lineage upon Notch activation. We found that freshly sorted CD9^High^ CMPs generated only megakaryocytic colonies (S12 Fig) but regenerated an increased number of bipotent and myeloid colonies after only two days culture on rDLL1 instead of IgGs (S12 Fig). In a similar attempt to isolate MK-committed HSCs, we found that, like CD9^High^ MEPs, sorted CD9^High^ LSKs generated pure megakaryocytic colonies of increased size after culture on rDLL1 instead of IgGs (S13 Fig). However, freshly sorted CD9^High^ LSKs generated only 15% of pure megakaryocytic colonies precluding the possibility to appreciate from this experiment if some of these MK-committed LSKs are also able to regenerate alternative lineages in response to Notch activation. We believe that addressing this question will require more sophisticated sorting protocols allowing the isolation of pure MK-committed LSK cells.

## Conclusion

In summary, our study demonstrates the pleiotropic effect of Notch signaling that stimulates the SCF-dependent proliferation of bipotent MEPs, favors their commitment towards the erythroid lineage, extends the proliferative potential of committed megakaryocytic progenitors while allowing a small fraction of them to reactivate latent alternative lineage potential. Such unexpected plasticity shared by a fraction of committed megakaryocytic progenitors, revealed here upon Notch stimulation, indicates that megakaryocytic priming does not necessarily impede commitment potential towards alternative lineages and may thus contributes to the known intriguing promiscuity between stem cells and megakaryocytic lineage.

## Supporting Information

S1 Fig MEP and LSK cells sorting. FACS diagrams illustrating the gating strategy used for the sorting of bone marrow (BM) MEPs expressing different levels of CD9 (**A**) and of bone marrow CD9^High^ LSK (**B**).
(PDF)

S2 Fig Transcript levels changes in total MEPs progeny after two days culture on rDLL1. Transcript levels were determined by qRT-PCR after two days cultures of MEP cells in the indicated conditions using β-actin as a reference. Results are presented as relative levels standardized to the culture condition on IgGs (means and standard deviations obtained from 3 independent cultures). Significant differences (p<0.05 in Student t-test) are indicated by asterisks.
(PDF)

S3 Fig The Amplification of bipotent and erythroid progenitors stimulated by Notch signaling is dependent on c-Kit/SCF signaling. Equal numbers of MEPs were cultured for two days in the presence or absence of SCF and their progenies were analyzed by colony assay as described in Fig 2. Results are expressed as fold variations of the number of each type of progenitors between Day 0 and Day 2 in presence (black boxes) or absence of SCF (white boxes). **A**: Bipotent colonies. **B**: Megakaryocytic colonies. **C**: Erythroid colonies. Means and standard deviations from three independent experiments. Significant variations (p<0.05 in Student t-test) are indicated by asterisks.
(PDF)

S4 Fig CD9 levels correlate with increased megakaryocytic potential of MEPs. Progeny analyses of equal numbers of sorted CD9^Low^, CD9^High^ and CD9^Med^ MEP subsets were performed by colony assays as described in Fig 2. **A**: FACS diagram showing the gating windows used for the sorting of MEP CD9^Low^, CD9^High^ and CD9^Med^ subsets; numbers correspond to the percentages of each subset. Dark filed diagram corresponds to control isotype labeling. **B:** Piled histograms showing the numbers of erythroid (Ery), megakaryocytic (Meg), erythro-megakaryocytic (Ery/Meg) and granulo-monocytic myeloid (G/M) colonies generated by the whole MEP population and by the CD9^Low^, CD9^Med^ and CD9^High^ MEP subsets immediately after sorting. Typical results from one of several experiments (see Day 0 in Figs 1, 2 and 3) but including CD9^Low^ subset.
(PDF)

S5 Fig Implication of Notch2 receptor in the changes of CD9^High^ MEPs progeny induced by rDLL1. Equal numbers of CD9^High^ MEP were cultured for 2 days on either IgGs or rDLL1 in the presence of a complete cocktail of myeloid cytokines and in the absence (W/o) or presence of either DAPT or 5 μg/mL of neutralizing Notch2 antibody (N2α) as indicated. The whole progenies generated in these different conditions were then analyzed by colony assays still in the presence of the same cocktail of myeloid cytokines as described in Fig 4. **A:** Histograms showing the percentages of different types of colonies counted including pure megakaryocytic colonies (MK total), bipotent erythro-megakaryocytic colonies (Ery/MK), pure erythroid (Ery) or myeloid (Myelo) colonies. **B:** Histograms showing the percentages of of pure megakaryocytic colonies containing either single (MK1), 2 to 8 (MK2-8) or more than 8 megakaryocytes (M>8). Results correspond to the means and standard deviations from two different counts of each of two duplicates from two independent experiments. Significant differences are indicated by asterisks (*, **, *** and **** for p values <0.05, <0.01, <0.001 and <0.0001 in Student t-test respectively; NS non significant). Note that all significant changes induced by rDLL1 (red braces), including the decrease in the percentages single megakaryocytes and total megakaryocytic colonies as well as the increase in the percentages of bipotent erythro-megakaryocytic and of the size of megakaryocytic colonies are all suppressed by both DAPT and Notch2 antibody (green braces) thus strongly supporting that all these changes are mediated by Notch2 receptor activation.
(PDF)

S6 Fig Notch stimulates the amplification of Kit^+^ progenitors from CD9^High^ MEPs. 1000 CD9^High^ MEPs were cultured for 5 days in the presence of a complete cocktail of myeloid cytokines (IL3, SCF, EPO, GM-CSF, TPO, Flt3L, IL11) in plate culture wells coated with either control IgG1 or recombinant rDLL1 in the presence or absence of DAPT as described in Fig 5. All cells present in each individual well were then collected, labeled with CD41 and c-Kit antibodies and the totality of labeled cells were analyzed by FACS thus allowing a semi-quantitative analysis of the different numbers of cells generated in the different culture conditions. Histograms show the total numbers of cells as well as the total numbers Kit^+^ cells (Means and standard deviations from 3 independent experiments). Significant differences (p value <0.05 in Student t-test) are indicated by asterisks.
(PDF)

S7 Fig Cell cycle analyses of CD9^High^ and CD9^Med^ MEPs before and after two days culture in the presence of absence of Notch ligand. **A**: FACS diagrams of CD9^Med^ MEPs (left panel) and CD9^High^ MEPs (right panel) after double labeling for DNA content (Propidium Iodide) and Ki67 expression immediately following their purification. Percentages indicate the proportion of diploid and Ki67 negative cells corresponding to classical G0 quiescent cells. Arrow indicates tetraploid cells expressing low levels of Ki67 suggesting quiescent G2/M cells that were present specifically in the CD9^High^ MEP subset. **B**: Cytospins of CD9^Med^ (left side) and CD9^High^ MEP cells (right side) after May Gründvald Giemsa staining. Arrow indicates binucleated cells specifically present in the MEP CD9^High^ subset. **C**: Histograms showing the repartition of CD9^Med^ and CD9^High^ cells in the G0/G1, S and G2/M phases of cell cycle before (Day 0) and after a two days culture (Day 2) on either IgG or rDLL1 (Mean results from two independent experiments are shown). **D**: Histogram showing the selective presence of 5% of binucleated cells in the MEP CD9^High^ subset.
(PDF)

S8 Fig Phase contrast images of the different types of colonies identified in single MEP progeny analyses reported in Fig 7. **A**: Pure megakaryocytic colony containing a small number of only mature megakaryocytes easily identified by their large size. **B**: Pure erythroid colony containing only mature erythroid cells easily identified by their small size. **C:** Mixed colony containing both erythroid and megakaryocytic cells. **D:** Pure myeloid colony containing large number of cells of intermediate size and irregular shape only. **E:** Multipotent colony containing erythroid, megakaryocytes and myeloid cells **F:** mixed colony containing erythroid and myeloid cells. FACS analyses showing the presence of CD11b^+^ cells were used as further confirmation of the myeloid potential (not shown). Note that pure megakaryocytic colonies were the only colonies generated by CD9^High^ MEP on IgGs (black rectangle) whereas the other types of colonies were generated only on rDLL1 (red rectangle).
(PDF)

S9 Fig rDLL1 increases Klf1 and Aurka transcript levels in CD9^High^ MEPs. Transcript levels were determined by qRT-PCR after two days cultures of CD9^High^ MEPs in the indicated conditions. Results are presented as relative levels standardized to β-actin (means and standard deviations from three independent cultures). Asterisks indicate statistically significant variations (p-value < 0.05 in Student t-test).
(PDF)

S10 Fig Retrospective transcriptome comparison between CD9^+^ and CD9^-^ MEP. **A**: Heatmap of genes upregulated (top) or downregulated (bottom) in CD9^+^ compared to CD9^-^ MEP. Analysis of transcriptome row data from the 64 single MEP recently published by Guo et al (Cell Stem cell. 2013; 13(4):492–505;) allowed us to identify and to virtually sort 16 and 48 single MEP expressing or not CD9 respectively. Heatmap presented here is limited to genes which mean expression levels were found statistically different between these virtually sorted CD9^+^ and CD9^-^ MEP subsets (p-value < 0.05 by Student t-test). Genes names and p-values are indicated on the left and right of the heatmap respectively. Mean expression levels of genes are indicated by numbers in table cells and further illustrated by increasing red color intensity. Note the marked difference between the contrasted differential expression of genes up-regulated compared to the modest differential expression of down regulated genes in CD9^+^ MEP. **B**: Expression profiles of genes differentially expressed between CD9^+^ and CD9^-^ MEP. Expression profiles of genes differentially expressed between CD9^+^ and CD9^-^ MEP were collected for stem cells (LT-HSC or ST-HSC), erythro-megakaryocytic bipotent (MKE), megakaryocytic (MKP) or erythrocytic (PreCFUE, CFUe and ProE) committed progenitors from Hemaexplorer murine dataset (GSE14833; http://servers.binf.ku.dk/bloodspot). Heatmap presented here corresponds to relative expression levels (mean of all specific probes levels for each given gene) normalized to the median expression level of the 7 different populations (number in table cells correspond to LOG(2) of normalized expression levels). Genes names are indicated on the left of the heatmap and are ordered separately for up-regulated and down regulated genes by decreasing expression levels in LT-HSC. Note that all genes up-regulated in CD9^+^ MEP correspond to genes displaying contrasted higher levels in LT-HSC and lower levels in committed erythrocytic progenitors, while most slightly down-regulated genes (DOWNb subset) display contrasted lower expression levels in LT-HSC and higher levels in committed erythrocytic progenitors.
(PDF)

S11 Fig Retrospective analysis of Fli-1, Klf1 and Aurka transcript levels from published transcriptomes of LSK stem cells and multipotent progenitors expressing endogenous Hes1 or exogenous ICN2. Normalized raw data corresponding to Hes1 (probe 1418102_at), Aurka (probe 1424511_at), Klf1 (probe 1418600_at), and Fli-1 (probes 1422024_at and 1433512_at) transcripts were collected from GEO dataset GSE46726. Histograms show the relative levels of Hes1 (**A**), Aurka (**B**), Klf1 (**C**) Fli-1 (**D)** and Fli1b (**E**) transcripts between LSK and multipotent progenitors (MP: Lin^-^Kit^+^Sca1^-^ population) expressing or not endogenous Hes1 or exogenous ICN2 (active intracellular fragment of Notch2 receptor) as indicated. Means and standard deviations from triplicates with statistically significant differences indicated by asterisks (*, **, and *** for p values <0.05, <0.01, and <0.001 in Student t-test respectively; NS non significant). Similar up regulations observed after two days culture of CD9^High^ MEPs on rDLL1 are indicated by red arrows respectively.
(PDF)

S12 Fig Freshly sorted CD9^High^ CMPs generate only megakaryocytic colonies but a strongly increased number of mixed colonies after a 2 days culture on rDLL1. 1000 CD9^High^ CMPs were cultured for two days in the presence of a complete cocktail of myeloid cytokines (IL3, SCF, EPO, GM-CSF, TPO, Flt3L, IL11) in plate culture wells coated with either control IgG1 or recombinant rDLL1 in the presence or absence of DAPT as indicated. Total numbers of bipotent erythro-megakaryocytic (E/Meg), pure erythroid (Ery) or megakaryocytic (Meg) progenitors present in the initial population (day 0) and after the two days culture in the different conditions were determined by colony assays performed in semi-solid medium in the presence of the same complete cocktail of cytokines as described for CD9^High^ MEPs in Fig 3. Piled histograms show the numbers of the different types of colonies generated by CD9^High^ CMPs before (Day 0) and after a two days culture on IgGs, rDLL1 or rDLL1 + DAPT (means of duplicate results from a single CMP preparation).
(PDF)

S13 Fig CD9^High^ LSK bone marrow cells generate larger megakaryocytic colonies when cultured on rDLL1 than on control IgG. Sorted CD9^High^ LSK cells (Lin^-^Sca1^+^Kit^+^) were analyzed by colony assay either directly or after a two days culture in the presence of a complete cocktail of myeloid cytokines (IL3, SCF, EPO, GM-CSF, TPO, Flt3L, IL11) in plate culture wells coated with either control IgG1 or recombinant rDLL1 in the presence or absence of DAPT as indicated. Total numbers and relative proportions of pure megakaryocytic colonies containing different numbers of megakaryocytes, mixed colonies containing megakaryocytes and other cells or only non megakaryocytic cells obtained in the different conditions were then recorded separately. **A:** Histograms showing the different numbers of each type of colonies obtained before (Day 0) and after the 2 days culture on either IgGs or rDLL1 with or without DAPT (data correspond to duplicates (#1 and #2) from a single experiment). **B:** Same data as in A but expressed as percentages of total colonies for each condition. **C, D, E, F:** Histograms showing the percentages of pure megakaryocytic colonies containing different numbers of megakaryocytes (from 1 to more than 10) at day 0 **(C)** and after two days culture on IgGs **(D)**, rDLL1 **(E)** or rDLL1 with DAPT **(F)**. Interestingly, the size of pure megakaryocytic colonies rapidly decreased after the two days culture on IgGs but this size decrease was strongly attenuated by culture on rDLL1 but not on rDLL1 and in the absence of DAPT. Taken together, these data strengthen our interpretation that Notch activation extends the proliferation potential of MK-committed progenitors by allowing them to performed additional divisions before terminal differentiation.
(PDF)

S1 Table Primer sequences used in qRT-PCR. (PDF)
