# Supplementary figures and images for: Notch Stimulates Both Self-Renewal and Lineage Plasticity in a Subset of Murine CD9High Committed Megakaryocytic Progenitors
Source: PLoS One. 2016 Apr 18;11(4):e0153860. doi: 10.1371/journal.pone.0153860 (PMC4835090; doi:10.1371/journal.pone.0153860)

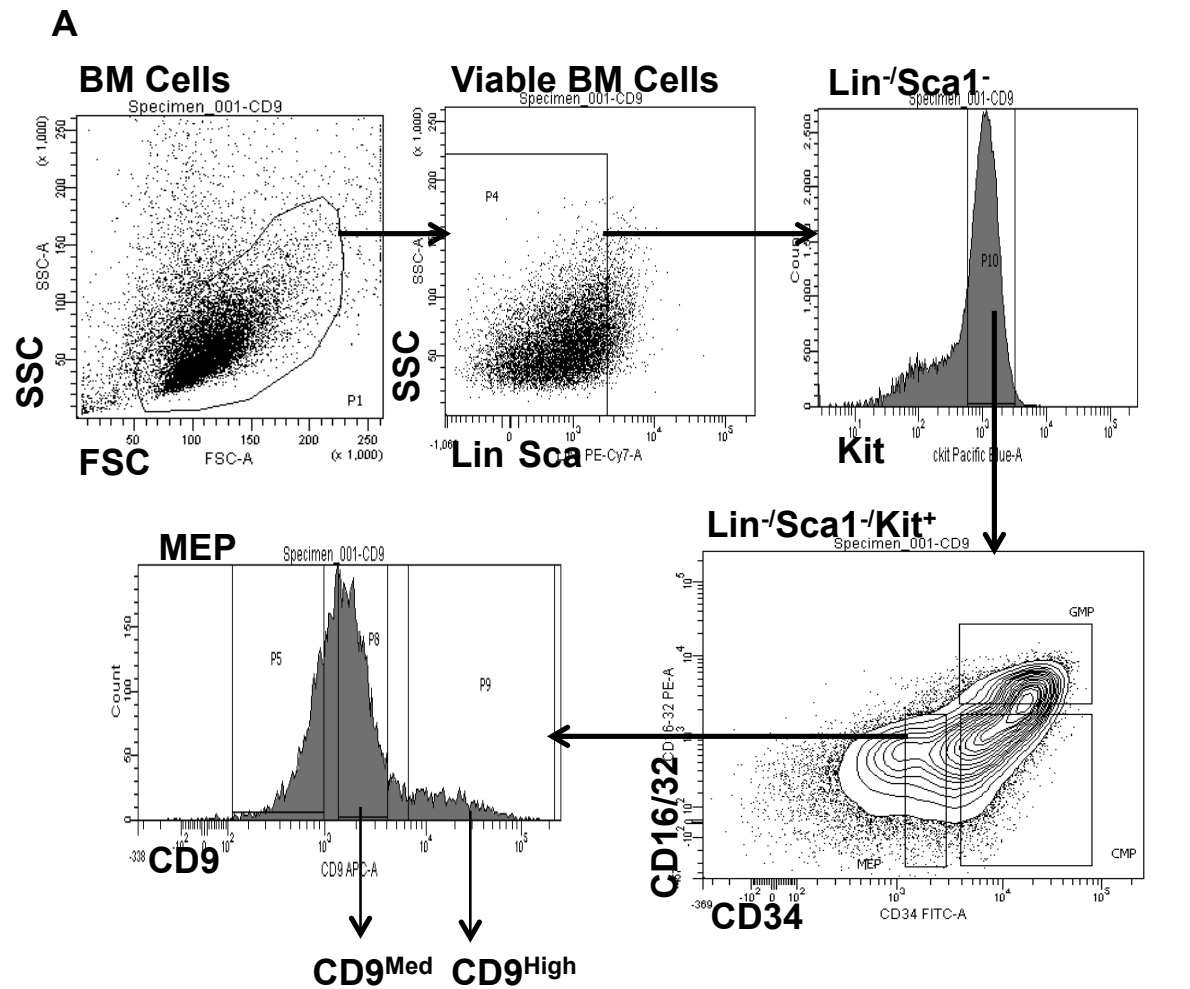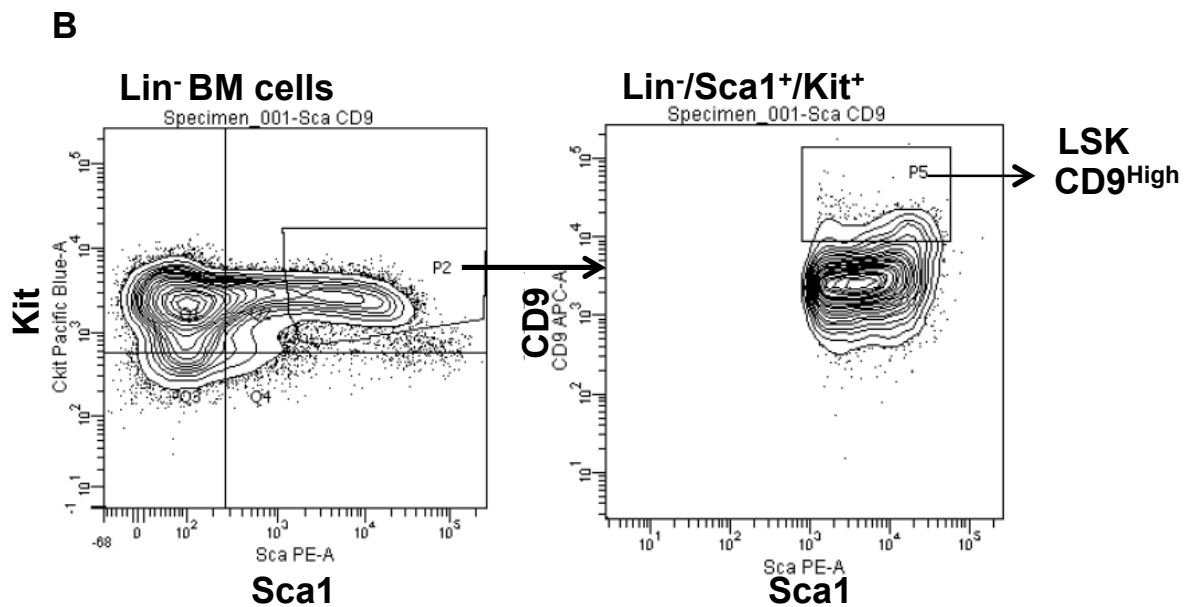

**S1 Figure**

Supplement: S1 Fig — FACS diagrams illustrating the gating strategy used for the sorting of bone marrow (BM) MEPs expressing different levels of CD9 (A) and of bone marrow CD9High LSK (B). (PDF) [file pone.0153860.s001.pdf]

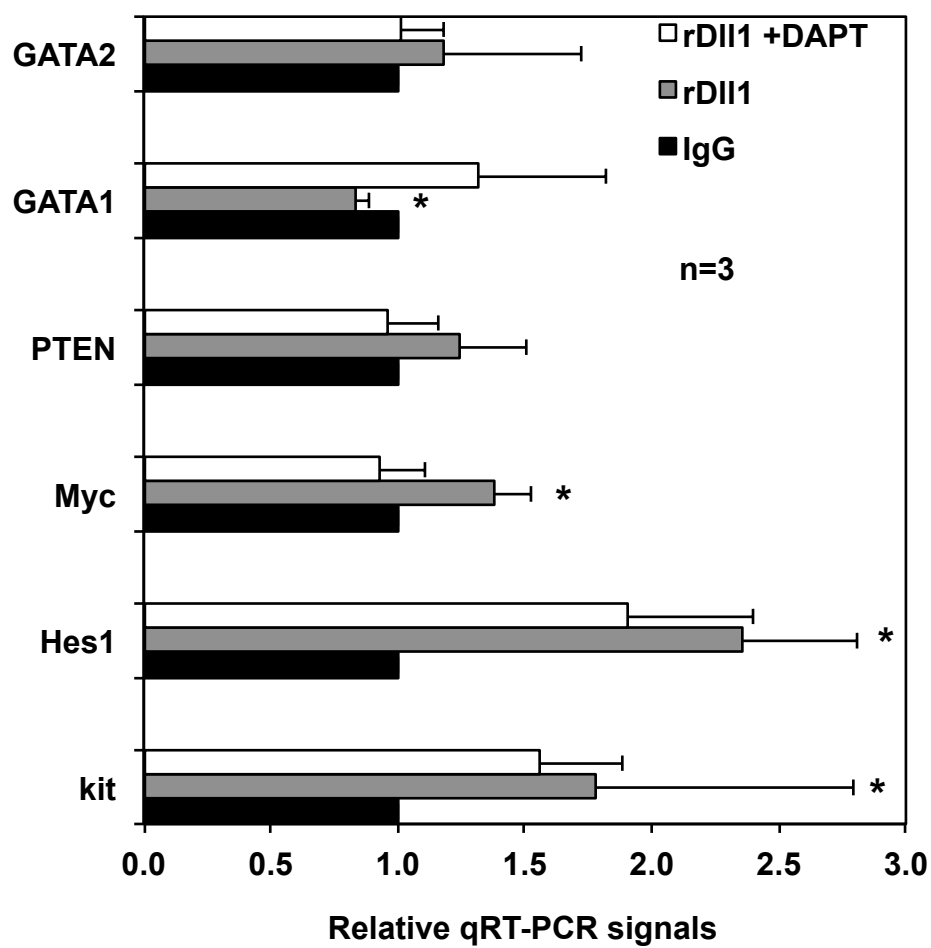

**S2 Figure**

Supplement: S2 Fig — Transcript levels were determined by qRT-PCR after two days cultures of MEP cells in the indicated conditions using β-actin as a reference. Results are presented as relative levels standardized to the culture condition on IgGs (means and standard deviations obtained from 3 independent cultures). Significant differences (p<0.05 in Student t-test) are indicated by asterisks. (PDF) [file pone.0153860.s002.pdf]

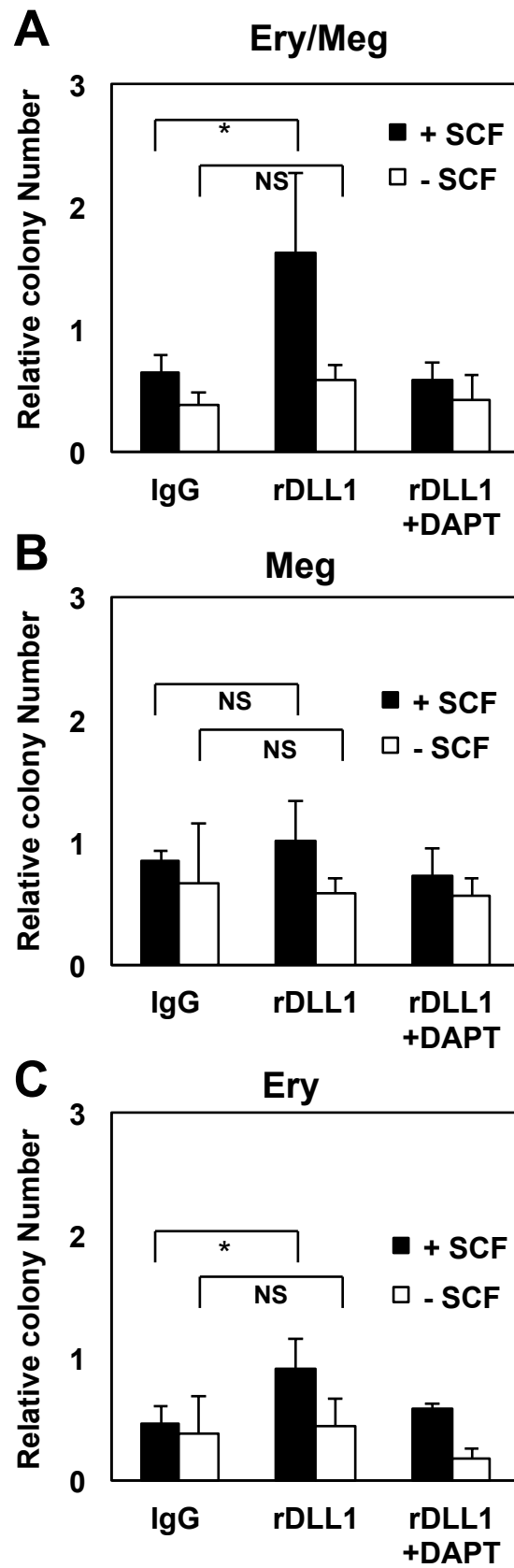

**S3 Figure**

Supplement: S3 Fig — Equal numbers of MEPs were cultured for two days in the presence or absence of SCF and their progenies were analyzed by colony assay as described in Fig 2. Results are expressed as fold variations of the number of each type of progenitors between Day 0 and Day 2 in presence (black boxes) or absence of SCF (white boxes). A: Bipotent colonies. B: Megakaryocytic colonies. C: Erythroid colonies. Means and standard deviations from three independent experiments. Significant variations (p<0.05 in Student t-test) are indicated by asterisks. (PDF) [file pone.0153860.s003.pdf]

**A**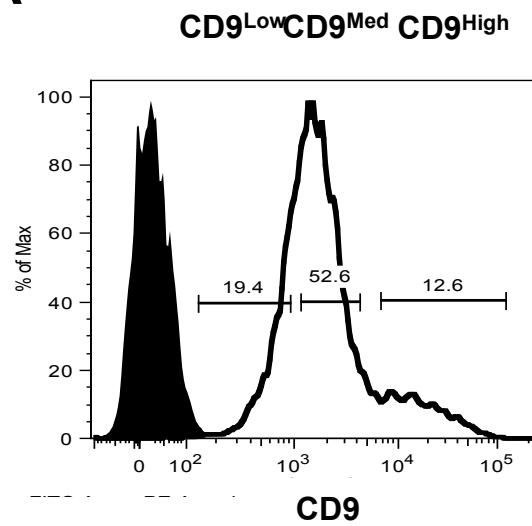**B**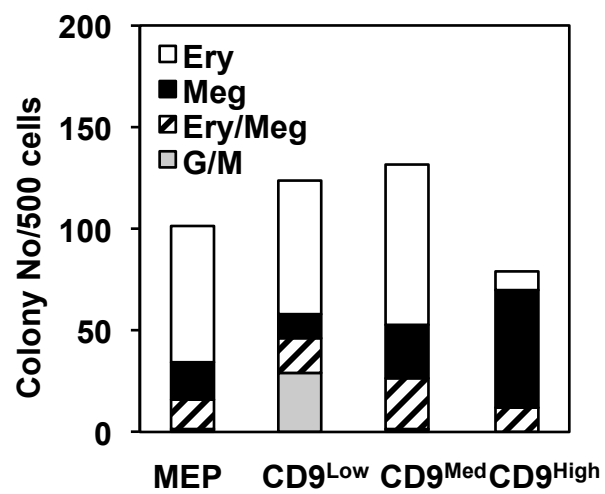**S4 Figure**

Supplement: S4 Fig — Progeny analyses of equal numbers of sorted CD9Low, CD9High and CD9Med MEP subsets were performed by colony assays as described in Fig 2. A: FACS diagram showing the gating windows used for the sorting of MEP CD9Low, CD9High and CD9Med subsets; numbers correspond to the percentages of each subset. Dark filed diagram corresponds to control isotype labeling. B: Piled histograms showing the numbers of erythroid (Ery), megakaryocytic (Meg), erythro-megakaryocytic (Ery/Meg) and granulo-monocytic myeloid (G/M) colonies generated by the whole MEP population and by the CD9Low, CD9Med and CD9High MEP subsets immediately after sorting. Typical results from one of several experiments (see Day 0 in Figs 1, 2 and 3) but including CD9Low subset. (PDF) [file pone.0153860.s004.pdf]

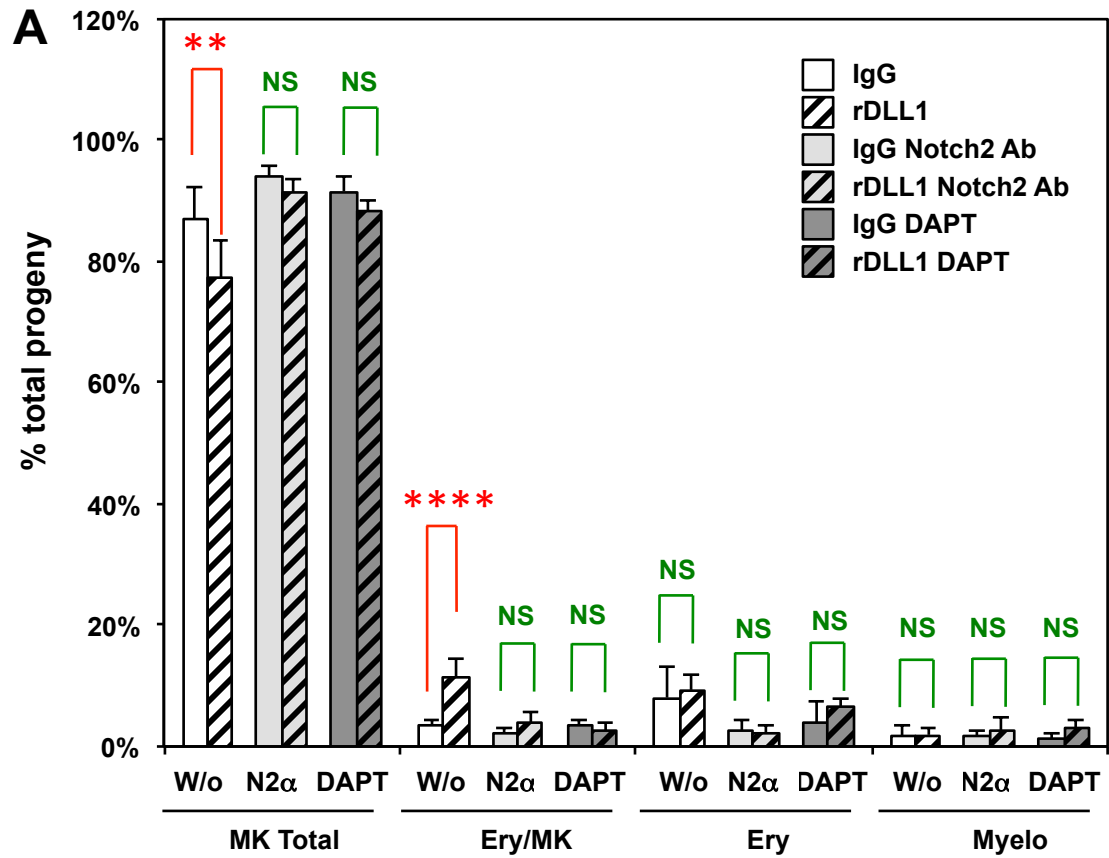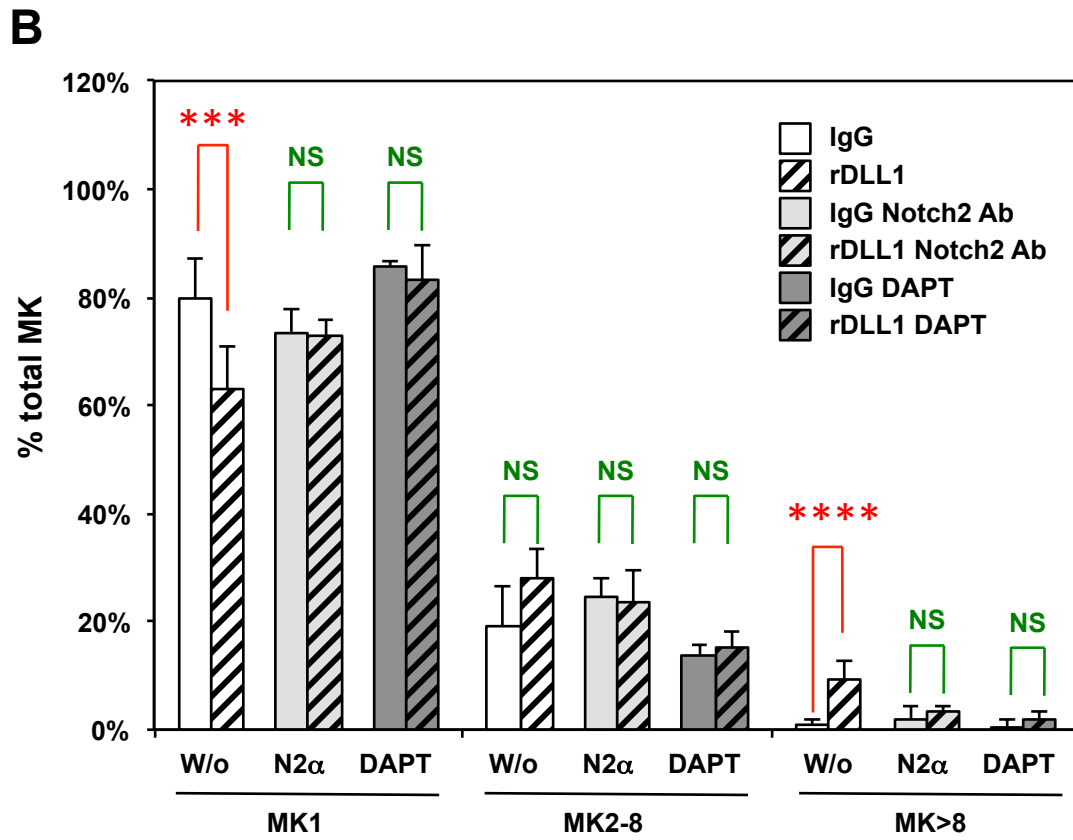

**S5 Figure**

Supplement: S5 Fig — Equal numbers of CD9High MEP were cultured for 2 days on either IgGs or rDLL1 in the presence of a complete cocktail of myeloid cytokines and in the absence (W/o) or presence of either DAPT or 5 μg/mL of neutralizing Notch2 antibody (N2α) as indicated. The whole progenies generated in these different conditions were then analyzed by colony assays still in the presence of the same cocktail of myeloid cytokines as described in Fig 4. A: Histograms showing the percentages of different types of colonies counted including pure megakaryocytic colonies (MK total), bipotent erythro-megakaryocytic colonies (Ery/MK), pure erythroid (Ery) or myeloid (Myelo) colonies. B: Histograms showing the percentages of of pure megakaryocytic colonies containing either single (MK1), 2 to 8 (MK2-8) or more than 8 megakaryocytes (M>8). Results correspond to the means and standard deviations from two different counts of each of two duplicates from two independent experiments. Significant differences are indicated by asterisks (*, **, *** and **** for p values <0.05, <0.01, <0.001 and <0.0001 in Student t-test respectively; NS non significant). Note that all significant changes induced by rDLL1 (red braces), including the decrease in the percentages single megakaryocytes and total megakaryocytic colonies as well as the increase in the percentages of bipotent erythro-megakaryocytic and of the size of megakaryocytic colonies are all suppressed by both DAPT and Notch2 antibody (green braces) thus strongly supporting that all these changes are mediated by Notch2 receptor activation. (PDF) [file pone.0153860.s005.pdf]

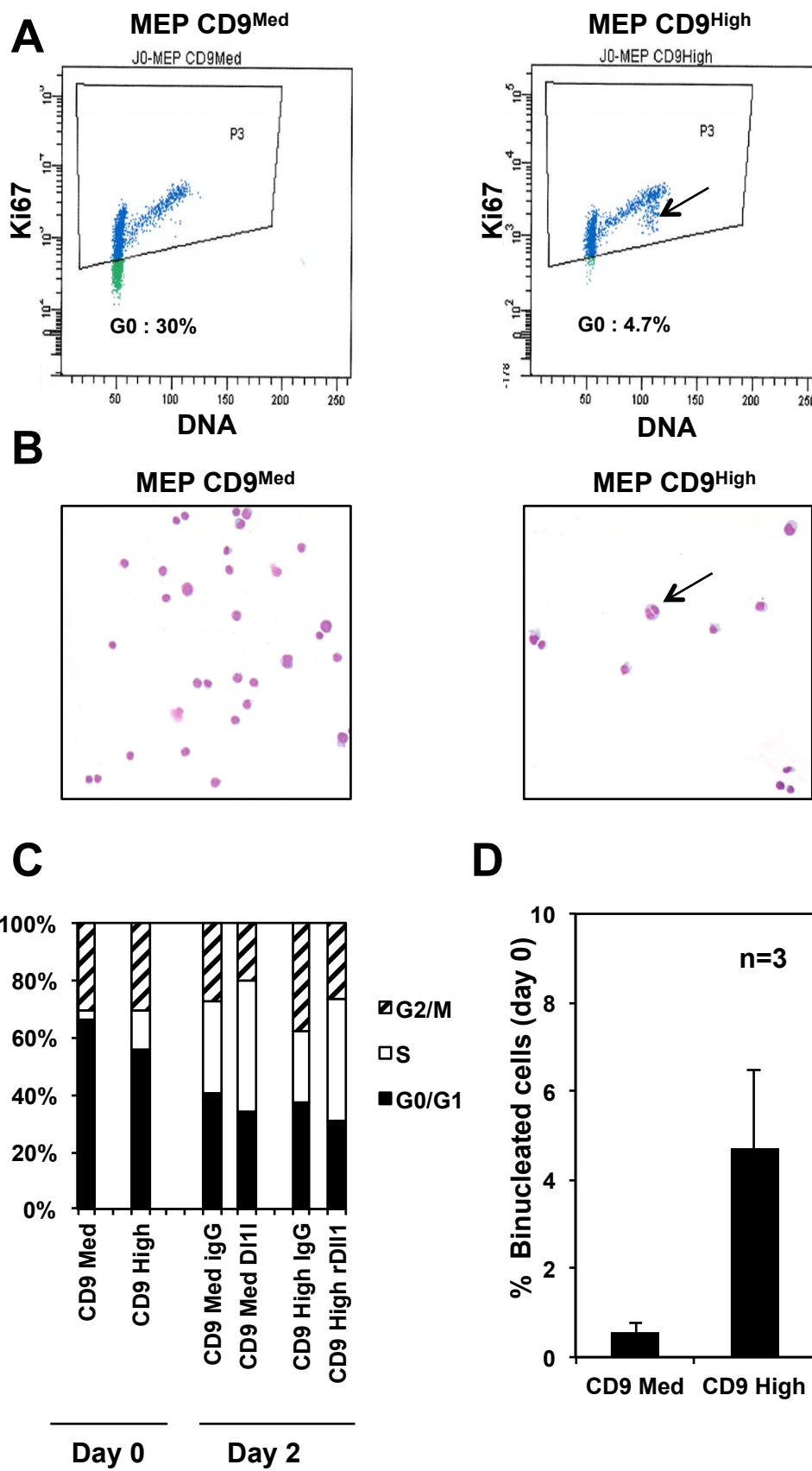

**S7 Figure**

Supplement: S7 Fig — A: FACS diagrams of CD9Med MEPs (left panel) and CD9High MEPs (right panel) after double labeling for DNA content (Propidium Iodide) and Ki67 expression immediately following their purification. Percentages indicate the proportion of diploid and Ki67 negative cells corresponding to classical G0 quiescent cells. Arrow indicates tetraploid cells expressing low levels of Ki67 suggesting quiescent G2/M cells that were present specifically in the CD9High MEP subset. B: Cytospins of CD9Med (left side) and CD9High MEP cells (right side) after May Gründvald Giemsa staining. Arrow indicates binucleated cells specifically present in the MEP CD9High subset. C: Histograms showing the repartition of CD9Med and CD9High cells in the G0/G1, S and G2/M phases of cell cycle before (Day 0) and after a two days culture (Day 2) on either IgG or rDLL1 (Mean results from two independent experiments are shown). D: Histogram showing the selective presence of 5% of binucleated cells in the MEP CD9High subset. (PDF) [file pone.0153860.s007.pdf]

**CD9<sup>High</sup> rDLL1**

**CD9<sup>High</sup> IgG**

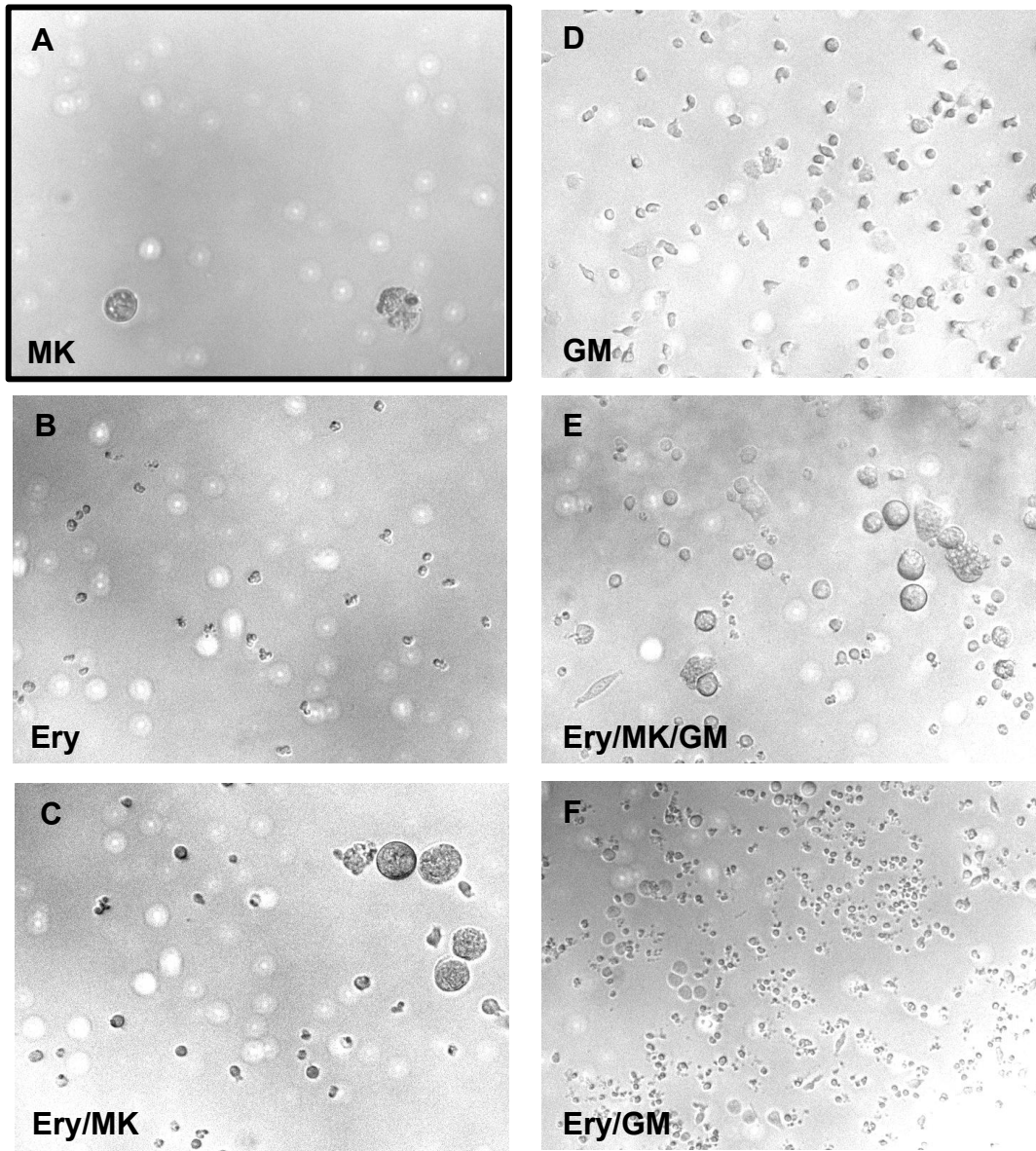

**S8 Figure**

Supplement: S8 Fig — A: Pure megakaryocytic colony containing a small number of only mature megakaryocytes easily identified by their large size. B: Pure erythroid colony containing only mature erythroid cells easily identified by their small size. C: Mixed colony containing both erythroid and megakaryocytic cells. D: Pure myeloid colony containing large number of cells of intermediate size and irregular shape only. E: Multipotent colony containing erythroid, megakaryocytes and myeloid cells F: mixed colony containing erythroid and myeloid cells. FACS analyses showing the presence of CD11b+ cells were used as further confirmation of the myeloid potential (not shown). Note that pure megakaryocytic colonies were the only colonies generated by CD9High MEP on IgGs (black rectangle) whereas the other types of colonies were generated only on rDLL1 (red rectangle). (PDF) [file pone.0153860.s008.pdf]

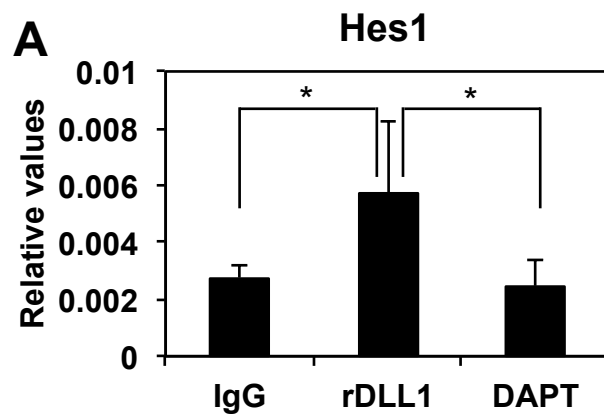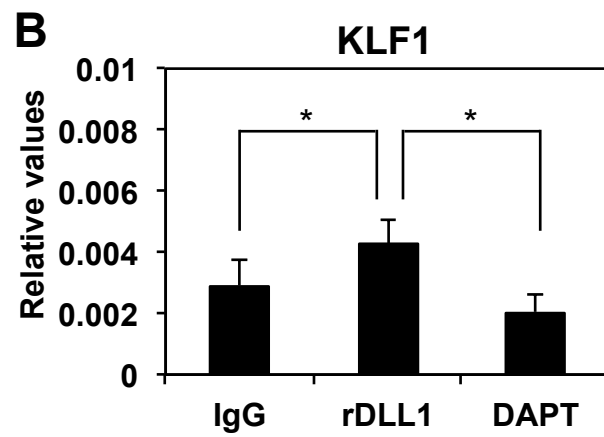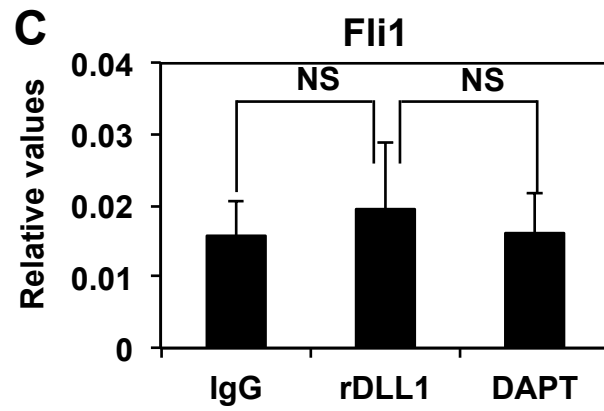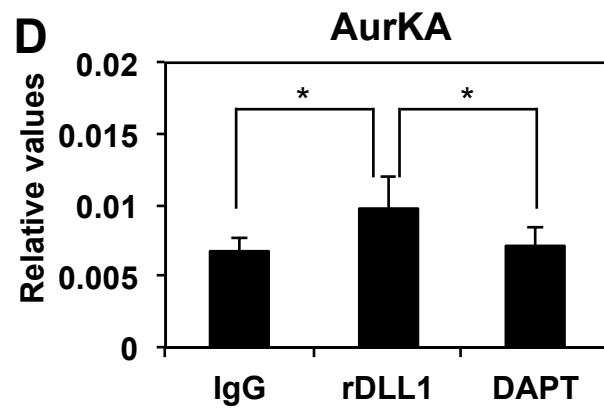

**S9 Figure**

Supplement: S9 Fig — Transcript levels were determined by qRT-PCR after two days cultures of CD9High MEPs in the indicated conditions. Results are presented as relative levels standardized to β-actin (means and standard deviations from three independent cultures). Asterisks indicate statistically significant variations (p-value < 0.05 in Student t-test). (PDF) [file pone.0153860.s009.pdf]

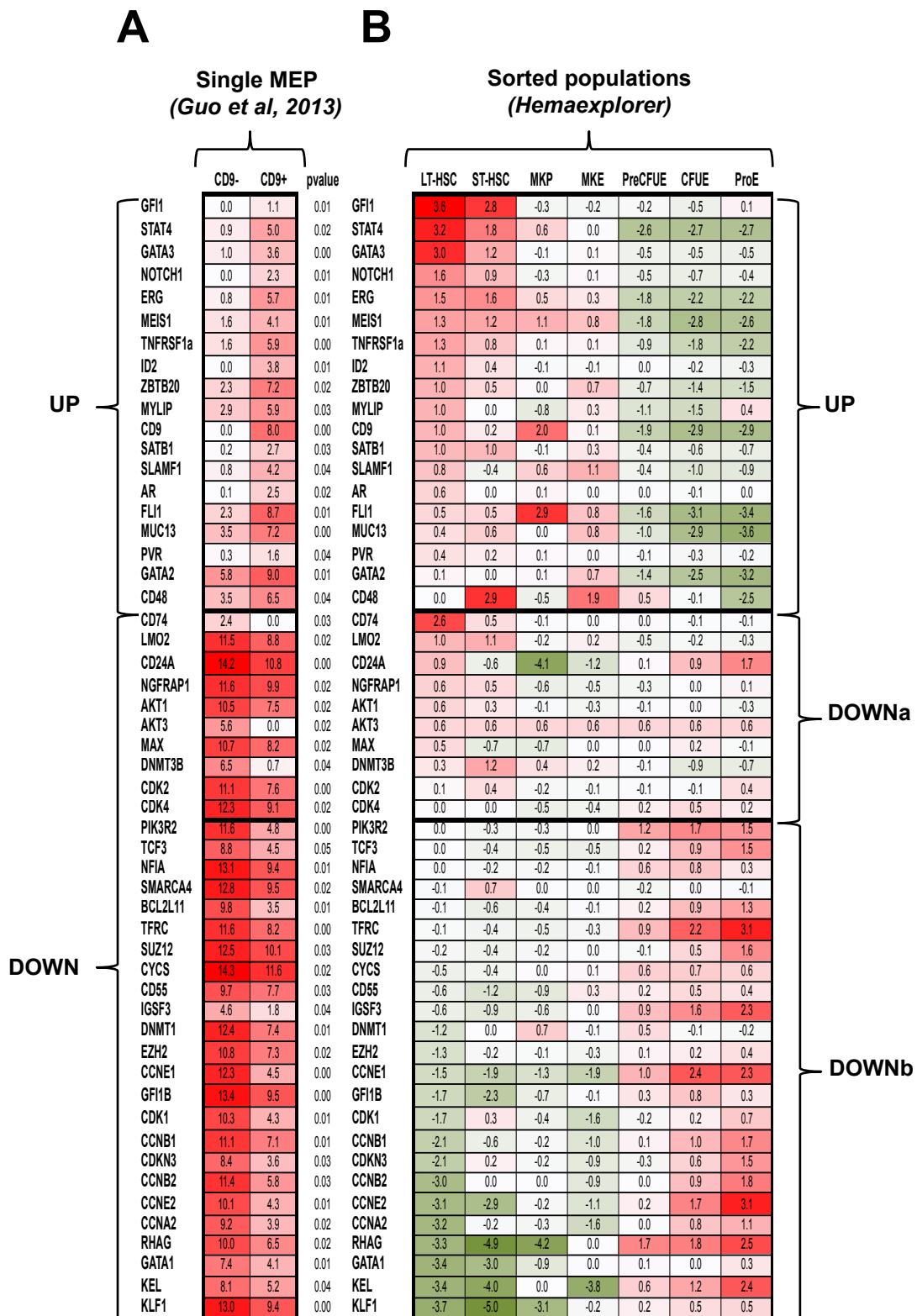

**S10 Figure**

Supplement: S10 Fig — A: Heatmap of genes upregulated (top) or downregulated (bottom) in CD9+ compared to CD9- MEP. Analysis of transcriptome row data from the 64 single MEP recently published by Guo et al (Cell Stem cell. 2013; 13(4):492–505;) allowed us to identify and to virtually sort 16 and 48 single MEP expressing or not CD9 respectively. Heatmap presented here is limited to genes which mean expression levels were found statistically different between these virtually sorted CD9+ and CD9- MEP subsets (p-value < 0.05 by Student t-test). Genes names and p-values are indicated on the left and right of the heatmap respectively. Mean expression levels of genes are indicated by numbers in table cells and further illustrated by increasing red color intensity. Note the marked difference between the contrasted differential expression of genes up-regulated compared to the modest differential expression of down regulated genes in CD9+ MEP. B: Expression profiles of genes differentially expressed between CD9+ and CD9- MEP. Expression profiles of genes differentially expressed between CD9+ and CD9- MEP were collected for stem cells (LT-HSC or ST-HSC), erythro-megakaryocytic bipotent (MKE), megakaryocytic (MKP) or erythrocytic (PreCFUE, CFUe and ProE) committed progenitors from Hemaexplorer murine dataset (GSE14833; http://servers.binf.ku.dk/bloodspot). Heatmap presented here corresponds to relative expression levels (mean of all specific probes levels for each given gene) normalized to the median expression level of the 7 different populations (number in table cells correspond to LOG(2) of normalized expression levels). Genes names are indicated on the left of the heatmap and are ordered separately for up-regulated and down regulated genes by decreasing expression levels in LT-HSC. Note that all genes up-regulated in CD9+ MEP correspond to genes displaying contrasted higher levels in LT-HSC and lower levels in committed erythrocytic progenitors, while most slightly down-regulated genes (DOWNb su [file pone.0153860.s010.pdf]

# GSE46726

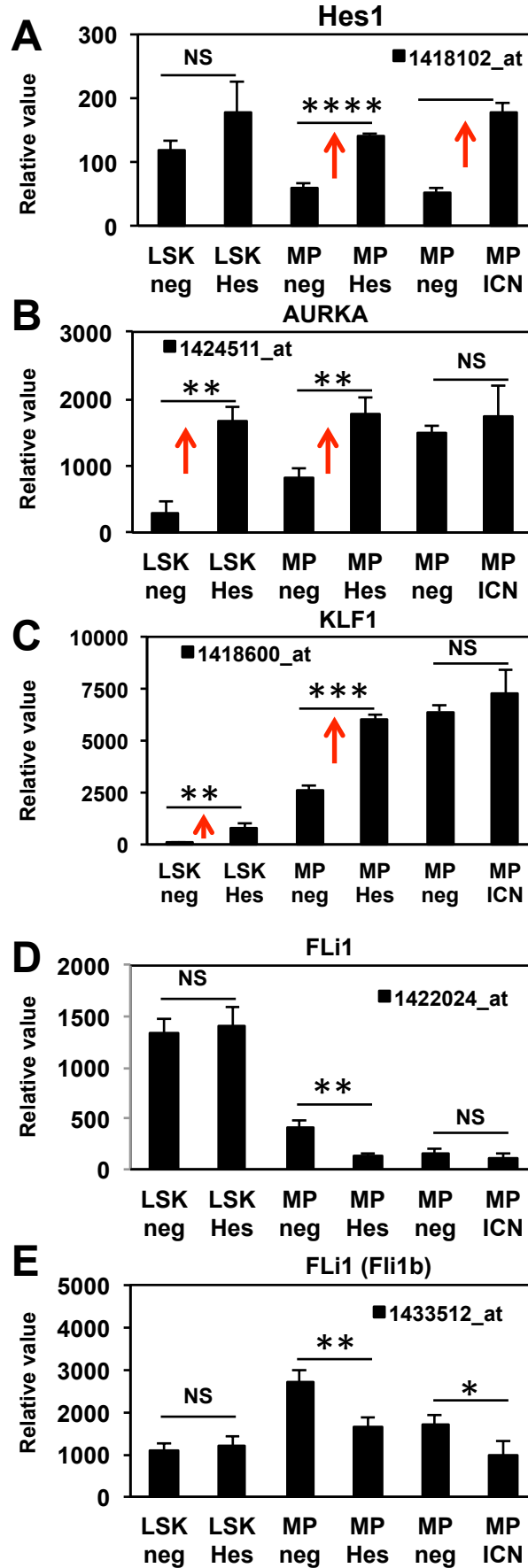

S11 Figure

Supplement: S11 Fig — Normalized raw data corresponding to Hes1 (probe 1418102_at), Aurka (probe 1424511_at), Klf1 (probe 1418600_at), and Fli-1 (probes 1422024_at and 1433512_at) transcripts were collected from GEO dataset GSE46726. Histograms show the relative levels of Hes1 (A), Aurka (B), Klf1 (C) Fli-1 (D) and Fli1b (E) transcripts between LSK and multipotent progenitors (MP: Lin-Kit+Sca1- population) expressing or not endogenous Hes1 or exogenous ICN2 (active intracellular fragment of Notch2 receptor) as indicated. Means and standard deviations from triplicates with statistically significant differences indicated by asterisks (*, **, and *** for p values <0.05, <0.01, and <0.001 in Student t-test respectively; NS non significant). Similar up regulations observed after two days culture of CD9High MEPs on rDLL1 are indicated by red arrows respectively. (PDF) [file pone.0153860.s011.pdf]

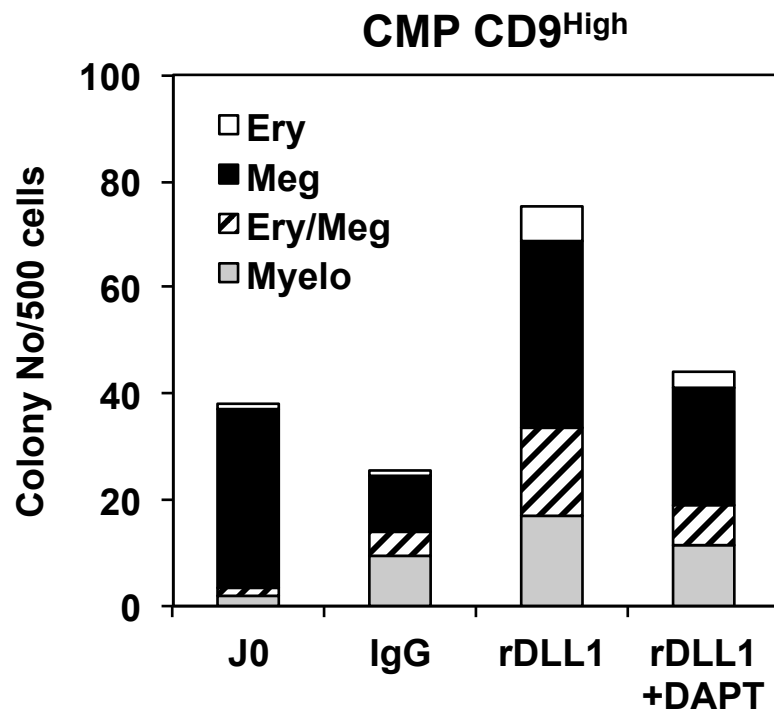

**S12 Figure**

Supplement: S12 Fig — 1000 CD9High CMPs were cultured for two days in the presence of a complete cocktail of myeloid cytokines (IL3, SCF, EPO, GM-CSF, TPO, Flt3L, IL11) in plate culture wells coated with either control IgG1 or recombinant rDLL1 in the presence or absence of DAPT as indicated. Total numbers of bipotent erythro-megakaryocytic (E/Meg), pure erythroid (Ery) or megakaryocytic (Meg) progenitors present in the initial population (day 0) and after the two days culture in the different conditions were determined by colony assays performed in semi-solid medium in the presence of the same complete cocktail of cytokines as described for CD9High MEPs in Fig 3. Piled histograms show the numbers of the different types of colonies generated by CD9High CMPs before (Day 0) and after a two days culture on IgGs, rDLL1 or rDLL1 + DAPT (means of duplicate results from a single CMP preparation). (PDF) [file pone.0153860.s012.pdf]

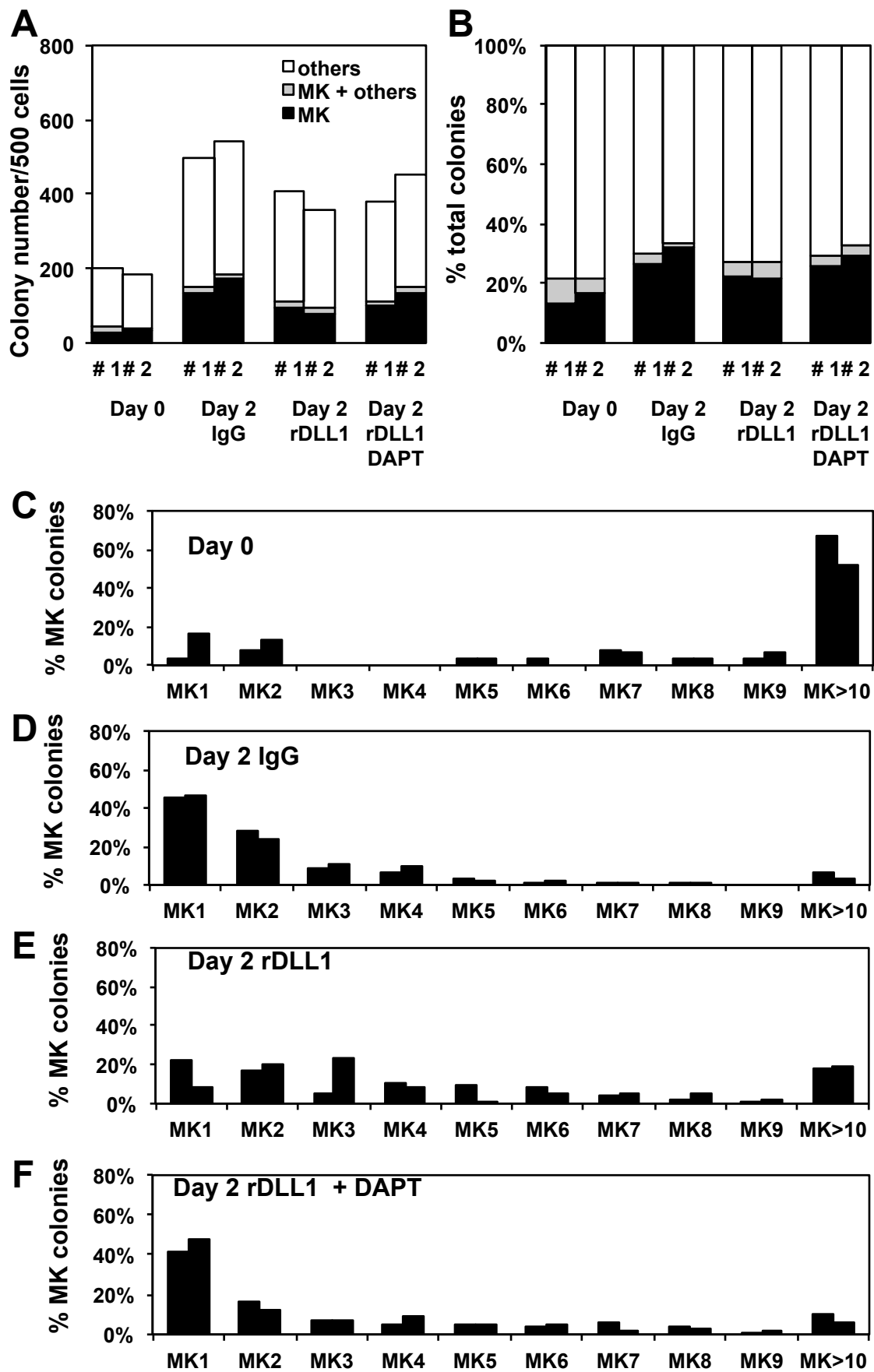

**S13 Figure**

Supplement: S13 Fig — Sorted CD9High LSK cells (Lin-Sca1+Kit+) were analyzed by colony assay either directly or after a two days culture in the presence of a complete cocktail of myeloid cytokines (IL3, SCF, EPO, GM-CSF, TPO, Flt3L, IL11) in plate culture wells coated with either control IgG1 or recombinant rDLL1 in the presence or absence of DAPT as indicated. Total numbers and relative proportions of pure megakaryocytic colonies containing different numbers of megakaryocytes, mixed colonies containing megakaryocytes and other cells or only non megakaryocytic cells obtained in the different conditions were then recorded separately. A: Histograms showing the different numbers of each type of colonies obtained before (Day 0) and after the 2 days culture on either IgGs or rDLL1 with or without DAPT (data correspond to duplicates (#1 and #2) from a single experiment). B: Same data as in A but expressed as percentages of total colonies for each condition. C, D, E, F: Histograms showing the percentages of pure megakaryocytic colonies containing different numbers of megakaryocytes (from 1 to more than 10) at day 0 (C) and after two days culture on IgGs (D), rDLL1 (E) or rDLL1 with DAPT (F). Interestingly, the size of pure megakaryocytic colonies rapidly decreased after the two days culture on IgGs but this size decrease was strongly attenuated by culture on rDLL1 but not on rDLL1 and in the absence of DAPT. Taken together, these data strengthen our interpretation that Notch activation extends the proliferation potential of MK-committed progenitors by allowing them to performed additional divisions before terminal differentiation. (PDF) [file pone.0153860.s013.pdf]
