# Supplementary material for: Notch Stimulates Both Self-Renewal and Lineage Plasticity in a Subset of Murine CD9High Committed Megakaryocytic Progenitors
Source: PLoS One. 2016 Apr 18;11(4):e0153860. doi: 10.1371/journal.pone.0153860 (PMC4835090; doi:10.1371/journal.pone.0153860)
Supplement: S1 Table — (PDF) [file pone.0153860.s014.pdf]

Target	Forward Primer	Reverse Primer
**β-Actin**	5’-TGGGAATGGGTCAGAAGGACTC-3’	5’-CTGGGTCATCTTTTCACGGTTG-3’
**Hes1**	5’-CTACCCCAGCCAGTGTCAAC-3’	5’-CGCCTCTTCTCCATGATAGG-3’
**Gata1**	5’-TTCTTCCACTTCCCCAAATG-3’	5’-AGGCCCAGCTAGCATAAGGT-3’
**Gata2**	5’-GAATGGACAGAACCGGCC-3’	5’-AGGTGGTGGTTGTCGTCTGA-3’
**Pten**	5’-AATTCCCAGTCAGAGGCGCTATGT-3’	5’-GATTGCAAGTTCCGCCACTGAACA-3’
**cMyc**	5’-TCCTGTACCTCGTCTGATTCC-3’	5’-CTCTTCTCCACAGACACCACATC-3’
**c-Kit**	5’-GGGCTAGCCAGAGACATCAG-3’	5’-AGGAGAAGAGCTCCCAGAGG-3’
**Fli-1**	5’-CCAACGAACGGAGAGTCTTG-3’	5’-TTCCCTGAGGTAACTGAGGTGC-3’
**Klf1**	5’-CGGGAAGAGCTACACCAAGA-3’	5’-GAGCGAACCTCCAGTCACAG-3’
**Aurka**	5’-TCTAGAATATGCGCCCCTTG-3’	5’-AGCGTTTGCCAACTCAGTG-3’

S1 Table. Primer sequences used in qRT-PCR
