# Supplementary material for: Notch Stimulates Both Self-Renewal and Lineage Plasticity in a Subset of Murine CD9High Committed Megakaryocytic Progenitors
Source: PLoS One. 2016 Apr 18;11(4):e0153860. doi: 10.1371/journal.pone.0153860 (PMC4835090; doi:10.1371/journal.pone.0153860)
Supplement: S6 Fig — 1000 CD9High MEPs were cultured for 5 days in the presence of a complete cocktail of myeloid cytokines (IL3, SCF, EPO, GM-CSF, TPO, Flt3L, IL11) in plate culture wells coated with either control IgG1 or recombinant rDLL1 in the presence or absence of DAPT as described in Fig 5. All cells present in each individual well were then collected, labeled with CD41 and c-Kit antibodies and the totality of labeled cells were analyzed by FACS thus allowing a semi-quantitative analysis of the different numbers of cells generated in the different culture conditions. Histograms show the total numbers of cells as well as the total numbers Kit+ cells (Means and standard deviations from 3 independent experiments). Significant differences (p value <0.05 in Student t-test) are indicated by asterisks. (PDF) [file pone.0153860.s006.pdf]

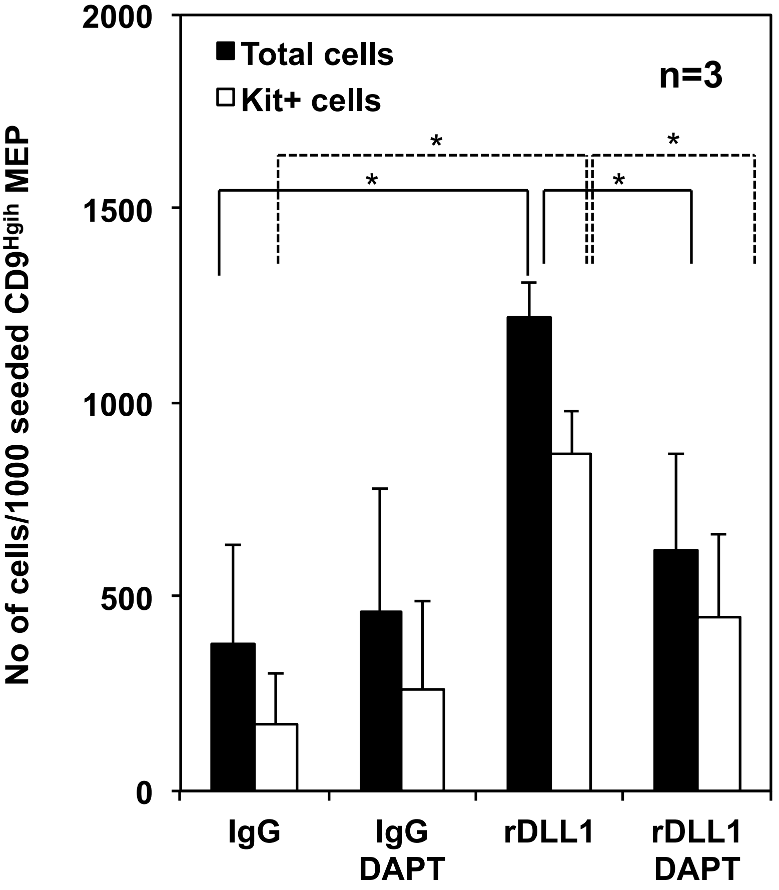
2000
1500
1000
500
0
Total cells
Kit+ cells
n=3
*
*
*
*
No of cells/1000 seeded CD9Hgih MEP
IgG
IgG DAPT
rDLL1
rDLL1 DAPT

S6 Figure
